# Social inequalities in patient outcomes after total hip replacement surgery for osteoarthritis in England: A population-based cohort study of the National Joint Registry

**DOI:** 10.1371/journal.pmed.1004870

**Published:** 2026-02-02

**Authors:** Rita Patel, Erik Lenguerrand, Yoav Ben-Shlomo, Jonathan French, Amar Rangan, Robin Brittain, Kevin Deere, Adrian Sayers, Ashley W. Blom, Michael R. Whitehouse, Andrew Judge

**Affiliations:** 1 Musculoskeletal Research Unit, Translational Health Sciences, Bristol Medical School, University of Bristol, Southmead Hospital, Bristol, United Kingdom; 2 National Institute for Health Research Biomedical Research Centre at University Hospitals Bristol and Weston NHS Foundation Trust and the University of Bristol, Bristol, United Kingdom; 3 Population Health Sciences, Bristol Medical School, University of Bristol, Bristol, United Kingdom; 4 The National Institute for Health and Care Research Applied Research Collaboration West (NIHR ARC West) at University Hospitals Bristol and Weston NHS Foundation Trust, Bristol, United Kingdom; 5 Department of Health Sciences & Hull York Medical School, University of York, Heslington, York, United Kingdom; 6 National Joint Registry for England, Wales, Northern Ireland and the Isle of Man, London, United Kingdom; 7 Nuffield Department of Orthopaedics, Rheumatology and Musculoskeletal Sciences, Nuffield Orthopaedic Centre, University of Oxford, Oxford, United Kingdom,; 8 Faculty of Medicine, Dentistry & Health, University of Sheffield, Sheffield, United Kingdom; Université Paris Cité UFR de Médecine: Universite Paris Cite UFR de Medecine, FRANCE

## Abstract

**Background:**

Reducing health inequalities is of national importance. Total hip replacement (THR) is a commonly used elective surgical procedure. Few studies have examined area-level inequalities for a wide range of outcomes following THR. The aim of this study is to compare area-level socioeconomic differences in outcomes following primary THR surgery for osteoarthritis in England.

**Methods and findings:**

This is a population-based prospective cohort study of the National Joint Registry (NJR). Data from the NJR were linked to national mortality, Hospital Episode Statistics and Patient Reported Outcome Measures (PROMs) databases for England from 2007 to 2017 with follow-up to 2023 for outcomes, for patients aged 50 years and over with osteoarthritis. Outcomes of 90-day mortality; 5-year revision rate; 6-month health complications; 1-year rehospitalisation and reoperation for orthopaedic indications; and patient-reported Oxford Hip Score (OHS), post-THR surgery were examined by area-level Index of Multiple Deprivation quintiles. Modified Poisson regression was adjusted for patient age, sex, body mass index, pre-operative physical state and comorbidity.

Among 448,184 patients with primary THR, mean age was 70 years (standard deviation: 9 years) and 61% were women. Patients from the most deprived group were more likely to die within 90 days of the operation compared to the least deprived group (adjusted rate ratio, RR: 1.25 (95% confidence interval (CI) [1.07, 1.46]); adjusted risk difference, RD: 9 (95% CI [2, 16]) per 10,000. Similarly, those from the most deprived group were more likely to experience complications (RR: 1.26 (95% CI [1.21, 1.32]); RD: 1.14% (95% CI [0.92, 1.36])); be rehospitalised (RR: 1.16 (95% CI [1.14, 1.19]; RD: 2.78% (95% CI [2.39, 3.17])) or reoperated (RR: 1.23 (95% CI [1.13, 1.33]); RD: 0.31% (95% CI [0.19, 0.44])) and report poorer OHS (adjusted score: −2.97 (95% CI [−3.10, −2.84]) *N* = 200,522). There was no variation by deprivation level for THR revision rates at 5 years (RR: 1.02 (95% CI [0.94, 1.10]); RD: 0.02% (95% CI [−0.10, 0.15])). The main study limitations are the lack of complete PROMs data, and the exclusion of self-funded patients or those with private insurance for THR procedures in independent hospitals.

**Conclusions:**

Inequalities in several outcomes after THR are present in England by area-level deprivation. These findings are useful to inform shared decision-making for patients deciding whether to undergo hip replacement and to benchmark the effectiveness of policies which aim to reduce health inequalities following THR.

## Author summary

**Why was this study done?:**

- Reducing health inequalities in the UK National Health Service (NHS) is of national importance.
- Total hip replacement (THR) is a commonly used elective surgical procedure.
- Few studies report area-level inequalities for a wide range of outcomes following THR, including patient-reported measures, in a national cohort using linked data over a long period.

**What did the researchers do and find?:**

- Using the largest national orthopaedic registry in the world, data for patients who received primary THR surgery over 11 years, were linked to hospital admissions, to examine the socioeconomic differences in a wide range of patient outcomes.
- This study found evidence of socioeconomic differences in complications, rehospitalisations and patient-reported wellbeing after surgery, with those from more deprived areas having worse outcomes, these differences were weaker for short-term mortality and reoperations, and not seen for 5-year revision rates.
- However, all groups gained equally in terms of patient-reported outcomes albeit from different pre-operative starting points.

**What do these findings mean?:**

- These findings are useful measures to inform shared decision-making for patients choosing whether to undergo hip replacement, and to benchmark the effectiveness of policies to reduce inequalities in the care pathways for the provision of THR.
- An important limitation of the study is that not all patients had complete information about how they felt before and after surgery. In addition, the experiences of people who paid privately or used private insurance for their hip replacement in non-NHS hospitals were not included.

## Abstract 3

Rita Patel and colleagues compare area-level socioeconomic differences in multiple outcomes, such as 90-day mortality and 5-year revision rates, among patients aged 50 years and over who underwent primary total hip replacement surgery for osteoarthritis in England.

## Introduction

The UK National Health Service (NHS) Long Term Plan expresses concern over growing inequalities in an expanding and ageing population [1]; these concerns are highlighted in major reviews [2,3] and are developed in the National Healthcare Inequalities Improvement Programme [4]. Furthermore, since the COVID pandemic there has been a call to reduce health inequalities in “The Build Back Fairer” review [5]. Total hip replacement (THR) is one of the most common elective procedures. The mean NHS cost of performing primary THR for the year of surgery (including primary, outpatient and inpatient hospitalisation) in 2017 was £9,295 [6]. In England, socioeconomic inequalities in the provision of THR have been reported and these have remained constant over time, with those from the most deprived groups less likely to receive treatment, despite greater need [7]. However, there is uncertainty regarding inequalities in the wide range of outcomes that are important to patients and stakeholders following THR in England. Previous studies examining area-level deprivation have reported mixed findings for outcomes following THR. Some have found higher short-term mortality associated with greater deprivation [8,9], while others have found no such relationship [8,10–12]. Similarly, evidence for an association between deprivation and revision risk [11,13] or postoperative complications [8,11,14] has generally been weak or absent, although findings for dislocation risk [8,9,15] have been inconsistent. Mixed results have also been reported for patient-reported outcomes, including the Oxford Hip Score (OHS) [8,9,16].

The aim of this study is to compare area-level socioeconomic differences for multiple outcomes, following primary THR surgery for osteoarthritis in England from 2007 to 2017 with follow-up to 2023, among patients aged 50 years and over. Using data from the National Joint Registry (NJR), the largest national orthopaedic registry in the world, data were linked to national mortality, Hospital Episode Statistics and Patient Reported Outcome Measures (PROMs) databases.

## Methods

### Ethics approval and consent to participate

With support under Section 251 of the NHS Act 2006, the Ethics and Confidentiality Committee (ECC) (now the Health Research Authority Confidentiality Advisory Group) allows the NJR to collect patient data where consent is indicated as “Not Recorded.” Before Personal Data and Sensitive Personal Data are recorded, express written patient consent is provided. The NJR records patient consent as either “Yes,” “No,” or “Not Recorded.” The NJR research committee approved this analysis, and the NHS Health Research Authority tool guidance dictates that the secondary use of such data for research does not require approval from a research ethics committee.

### Data sources

This is an observational study using prospectively collected anonymised data for patients in the NJR. The NJR registers mandatory data from surgeons and their hospitals regarding all joint replacement activity for hips, knees, shoulders, elbows and ankles whether they were funded by the NHS or independently in England, Wales, Northern Ireland, the Isle of Man and Guernsey. The NJR passes details of patients with primary or revision joint replacement procedures to the NHS Personal Demographics Service which provides dates of death from the Office for National Statistics if the NHS number is traceable. Information on the area of residence of patients, as defined by the 2011 census Lower Layer Super Output Areas (LSOAs) were collected. The relative level of deprivation of patient’s area of residence was obtained using the Index of Multiple Deprivation (IMD) version 2015 [17]. Each LSOA was linked to an IMD deprivation score and ranked according to deprivation deciles. IMD deciles were calculated by ranking the 32,844 small areas in England from most deprived to least deprived and splitting them into 10 equal-sized groups [17]. The exposure was IMD quintiles derived from the IMD deciles, with deciles 1 and 2 producing quintile 1 (Q1)—associated with the 20% most deprived LSOAs nationally—up to deciles 9 and 10 creating quintile 5 (Q5), representing the 20% of least deprived LSOAs.

Patients eligible for inclusion received a primary THR between 1 January 2007 and 31 December 2017 for the sole indication of osteoarthritis (which was the most common operative indication 87.9%) [18], and were aged 50 years or more, operated on and living at the time of surgery in England as indicated by LSOA, and had follow-up data to 2023 for outcomes. Patients who had metal-on-metal implants or simultaneous (performed on the same day) bilateral THRs were excluded. Those included had a first unilateral THR procedure only, either (i) a unilateral THR or (ii) a non-synchronous bilateral THR performed on different dates (in which case only the outcomes for the first replacement were examined).

The NJR data were linked to routinely collected Hospital Episode Statistics (HES) Admitted Patient Care and NHS England PROMs databases. NJR records could be linked to HES or PROMs if they took place in an NHS Trust or were funded by the NHS in England. The unit of analysis in this cohort was the individual.

### Outcomes

Outcomes examined following THR surgery were:

(i) **Cumulative 90-day all-cause mortality:** Date of death minus date of primary THR was calculated to determine mortality status at 90-days following this operation.
(ii) **Cumulative 5-year hip revision:** Date of first revision minus date of primary THR for the same joint were used to determine all-cause revision status at 5 years. The three most common reasons for revision were examined: any indication which included dislocation, infection, or aseptic loosening.
(iii) **Cumulative health complications recorded by 6 months:** Health complications at 6 months post-primary THR were determined using selected International Classification of Diseases (ICD10) codes recorded in 20 HES diagnosis fields for each admitted patient care episode. Complications were: (a) health-related: urinary tract infection; respiratory tract infection (including pneumonia); acute renal failure; acute myocardial infarction; stroke (excluding mini stroke); (b) surgery-related: complication of prosthesis; pulmonary embolism or deep vein thrombosis; surgical site infection; wound disruption; fracture after implant; and neurovascular injury (S1 Table). In addition, blood transfusion was included as a surgery-related complication and was determined using selected Classification of Interventions and Procedures (OPCS-4) codes recorded in 24 HES operation fields (S1 Table).
(iv) **Cumulative rehospitalisation and reoperation for an orthopaedic indication by 1 year:** Codes from existing publications were extracted and indicated on a comprehensive ICD10/OPCS-4 code list, these lists were reviewed and classified by an orthopaedic surgeon (JF). Rehospitalisation was determined by searching diagnosis fields for specific ICD10 codes (S2 Table) recorded in the year post-primary THR. Similarly, reoperation was determined by searching operation fields for specific OPCS-4 codes (S3 Table) in the year post-primary. Primary or revision hip admissions or operations recorded in the NJR database were excluded.
(v) **PROMs using the Oxford Hip Score (OHS):** OHS [19] for pain and function related to the affected hip was measured up to 6 months before (pre) and up to 12 months after primary THR (post) [20]. OHS was derived from 12 questions, related to hip pain and function domains, added to form an overall total, with subscale scores related to each domain (each utilising 6 questions) [19]. Each question is scored from 0 to 4 with 4 being the best outcome, with a maximum of 48 for the full score and 24 for the subscales [21]. The difference in the scores was used to determine the change in score from pre- to post-THR. The Minimal Clinically Important Difference (MCID) for the full score was set at five points [22]. MCID was dichotomised (0/1) as ‘Improved ≥5 points’ (coded as 0) versus ‘Not improved or worse <5 points’ (coded as 1), respectively, to allow presentation of findings with other dichotomous outcomes using a clinically relevant measure.

### Adjustment factors

Other variables included: sex (female, male); age group (50–54, 55–64, 65–74, 75+ years old); body mass index (BMI < 18.5 underweight, 18.5–24.9 normal, 25–29.9 overweight, ≥30 kg/m^2^ obese, unknown BMI); American Society of Anesthesiologists’ classification of pre-operative physical status (ASA grade P1-Fit and healthy, P2-Mild disease not incapacitating, P3-Incapacitating systemic disease, P4-Life threatening disease/P5-Expected to die within 24 h) [23]; and Royal College of Surgeon’s Charlson score (No comorbid conditions, Mild-one comorbid condition, Moderate-two comorbid conditions, Severe-three or more comorbid conditions) [24]. The Charlson score was calculated based on the presence of several chronic conditions, identified using ICD-10 codes at the hip replacement admission and all admissions in the preceding 3 years.

### Statistical analysis

Prevalence rates were calculated as percentage or per 10,000 persons for IMD groups, year of joint replacement and other patient characteristics. Modified Poisson regression models [25] with a log link function and robust standard errors to account for clustering of patients within hospitals were used to compare rates and estimate rate ratios (RRs), as well as to calculate absolute differences for risk differences (RDs). The number needed to harm (NNTH) were calculated as the reciprocal of the RD. The models were adjusted for patient characteristics measured at the primary operation: patient sex, age group, BMI, ASA grade and Charlson score as categorised above. As the association between age and many of the outcomes was non-linear, age was modelled using age groups to account for this non-linearity. Linear regression models were used with the continuous post-op OHS as the outcome variable, adjusting for the pre-operative OHS, IMD deprivation score as the primary exposure variable, and the patient characteristics of sex, age group, BMI, ASA grade and Charlson score.

Sensitivity analysis calculated adjusted RRs for: (1) the larger cohort of those that did not have linked HES data but for whom NJR outcomes of mortality and revision were available (adjusted for sex, age group, BMI and ASA grade at primary operation, but not Charlson score as this is derived from HES data); the main outcomes were additionally adjusted (2a) for pre-THR OHS for those patients who had PROMs scores reported; and (2b) for calendar year. Analyses were conducted using Stata version 18.5 statistical software (StataCorp, College Station, Texas). The RECORD (REporting of studies Conducted using Observational Routinely-collected health Data) guideline was followed to report the study (S1 Checklist) [26].

### Patient involvement

Patient representatives sit on the committee structure of the NJR. The research priorities of the NJR are identified and approved by patient representatives as part of this committee. One of the NJR patient representatives (RB), was involved in setting the research question and outcome measures, contributed to designing this work, discussing findings, and interpreting the results in a meaningful way for a lay audience. We are unable to disseminate results of this study directly to study participants due to the anonymous nature of the data. We plan to disseminate our findings to the NJR, via their communications team, to consultations relevant to the provision of joint replacement and to the general population through the local and national press.

## Results

**Fig 1:**
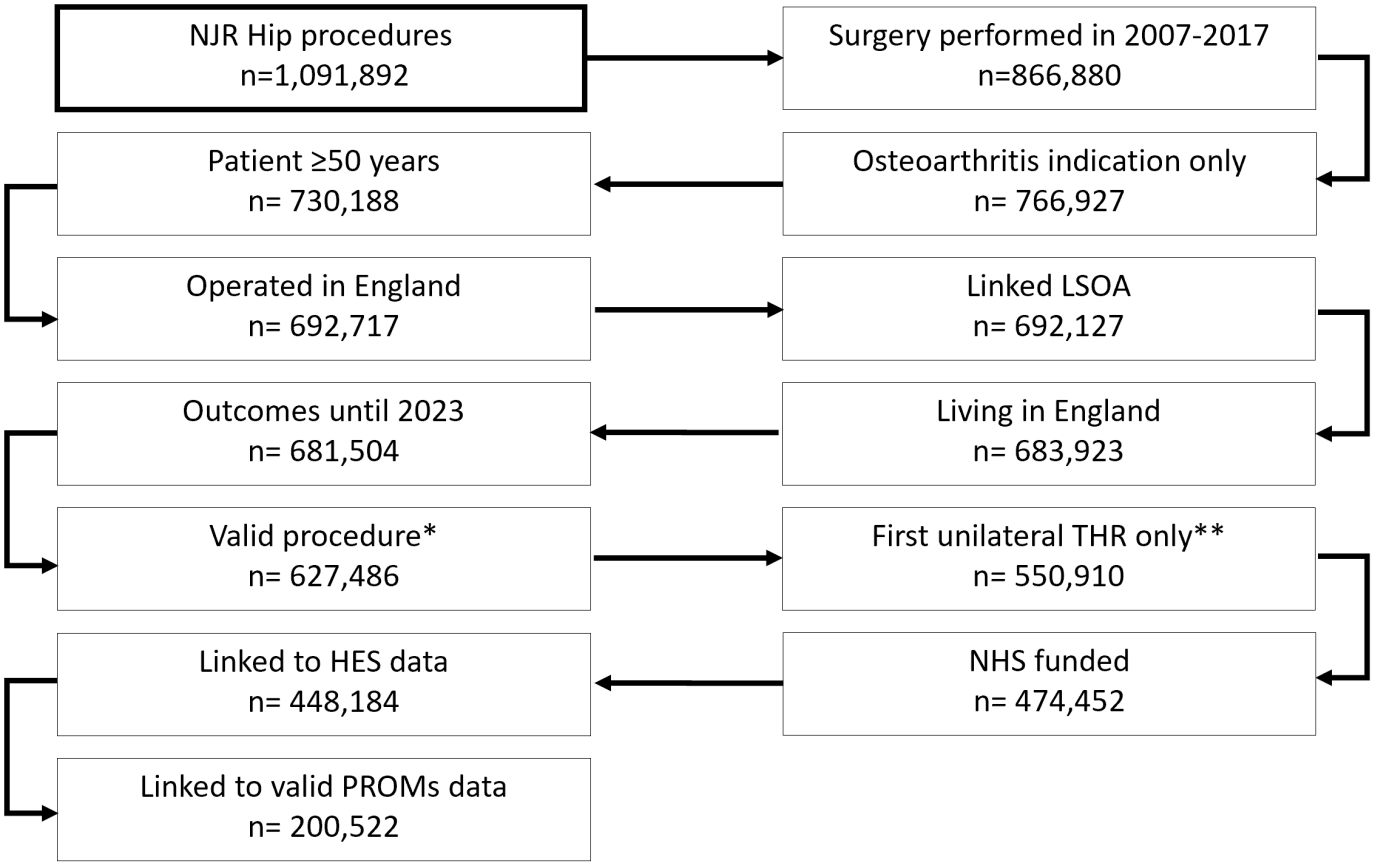
Flow of patients with hip procedures. * Metal-on-metal implants or simultaneous (performed on the same day) bilateral THRs excluded. ** First unilateral THR (i.e., unilateral THR or first non-synchronous [performed on different dates] bilateral THR). Abbreviations: HES, Hospital Episode Statistics; IMD, Index of Multiple Deprivation; LSOA, Lower Layer Super Output Area; n, number; NHS, National Health Service; NJR, National Joint Registry; PROMs, Patient Reported Outcome Measures; THR, Total hip replacement.

Of the 550,910 patients with a first unilateral THR (i.e., unilateral THR or first non-synchronous [performed on different dates] bilateral THR) that met the inclusion criteria, 76,458 (13.9%) could not be linked to HES data as they were recorded as independently funded. These patients had index THR operations undertaken in an independent hospital, i.e., not NHS funded and not treated in an NHS hospital, and hence not captured in HES data. A further 26,268 individuals (5.5%) were NHS-funded; however, their records could not be linked to HES data (Fig 1).

Among included patients (*N* = 448,184), the mean age at primary THR was 70 years (standard deviation: 9 years) and 61% were women (Table 1). Most patients (54%) were either overweight or obese, although a third (32%) of patients did not have a recorded BMI. Eighty-three percent of patients were ASA grade P1 (fit and healthy) or P2 (mild disease not incapacitating) at the primary operation. The number of people included in this analysis having THR surgery per year almost doubled from 26,928 to 48,484 from 2007 to 2017. Those in the least deprived group represented 24% of the cohort, with only 13% of patients in the most deprived group (Table 1). The national PROMs programme began in 2009; hence data were not collected at all in 2007/8 and were more likely to be collected in later years of the study as completion rates improved with time. Overall, those from the most deprived IMDs were less likely to have valid PROMs data in terms of completeness and within the time limits (48% had complete PROMs data among the least deprived IMD group versus 39% among the most deprived) *p* < 0.001 (S4 Table).

**Table 1:** Characteristics of patients at primary total hip replacement in England with NJR, linked HES and linked PROMs data (2007–2017).

Characteristics		NJR data		NJR linked HES data		NJR linked HES with valid PROMs data		Those missing valid PROMs data among 448,184 patients*
		*N*	%	*N* ^1^	%	*N* ^2^	%	%
Total	Patients	550,910	100	448,184	100	200,522	100	55
Sex	Female	335,319	61	272,974	61	119,768	60	56
	Male	215,591	39	175,210	39	80,754	40	54
Age (years)		(Mean = 70.3)	(SD = 9.1)	(Mean = 70.4)	(SD = 9.1)	(Mean = 69.9)	(SD = 8.8)	
Age at primary (years)	50–54	28,572	5	22,737	5	9,793	5	57
	55–64	120,445	22	96,418	22	44,852	22	53
	65–74	212,250	39	173,200	39	81,552	41	53
	75+	189,643	34	155,829	35	64,325	32	59
Body Mass Index at primary (kg/m^2^)	<18.5	3,159	1	2,471	1	1,040	1	58
	18.5 to 24.9	76,442	14	59,091	13	28,663	14	51
	25 to 29.9	151,867	28	122,466	27	62,089	31	49
	≥30	146,484	27	122,911	27	59,637	30	51
	Unknown BMI	172,958	31	141,245	32	49,093	24	65
ASA grade	P1—Fit and healthy	70,526	13	51,890	12	25,021	12	52
	P2—Mild disease not incapacitating	392,228	71	319,193	71	145,754	73	54
	P3—Incapacitating systemic disease	85,741	16	74,923	17	29,074	15	61
	P4—Life threatening disease/P5—Expected to die within 24hrs	2,415	<1	2,178	1	673	<1	69
Charlson score (morbidity)	None	NA	NA	289,327	65	131,399	66	55
	Mild	NA	NA	82,785	18	35,984	18	57
	Moderate	NA	NA	45,235	10	20,325	10	55
	Severe	NA	NA	30,837	7	12,814	6	58
Year of operation	2007	36,188	7	26,928	6	NA	NA	100
	2008	38,720	7	29,589	7	NA	NA	100
	2009	41,514	8	31,726	7	10,046	5	68
	2010	46,216	8	37,056	8	19,053	10	49
	2011	49,058	9	40,007	9	21,698	11	46
	2012	52,336	10	43,514	10	22,487	11	48
	2013	53,461	10	44,698	10	24,186	12	46
	2014	57,451	10	48,281	11	26,247	13	46
	2015	57,620	10	48,346	11	26,259	13	46
	2016	59,559	11	49,555	11	26,754	13	46
	2017	58,787	11	48,484	11	23,792	12	51
IMD	Q5-Least deprived	141,704	26	106,374	24	50,741	25	52
	Q4	136,834	25	108,614	24	50,174	25	54
	Q3	122,245	22	100,676	23	45,259	23	55
	Q2	88,286	16	76,521	17	32,612	16	57
	Q1-Most deprived	61,841	11	55,999	13	21,736	11	61

Figures may not add up to 100 percent due to rounding.

* Percentage calculated as: (Number missing/Total number) × 100, where Number missing is *N*^1^ − *N*^2^ and Total number is *N*^1^.

Abbreviations: ASA, American Society of Anesthesiologists’; BMI, Body Mass Index; HES, Hospital Episode Statistics; IMD, Index of Multiple Deprivation; N, number; NA, Not available; NJR, National Joint Registry; PROMs, Patient Reported Outcome Measures; Q, quintile; SD, standard deviation.

### Ninety-day all-cause mortality

**Fig 2:**
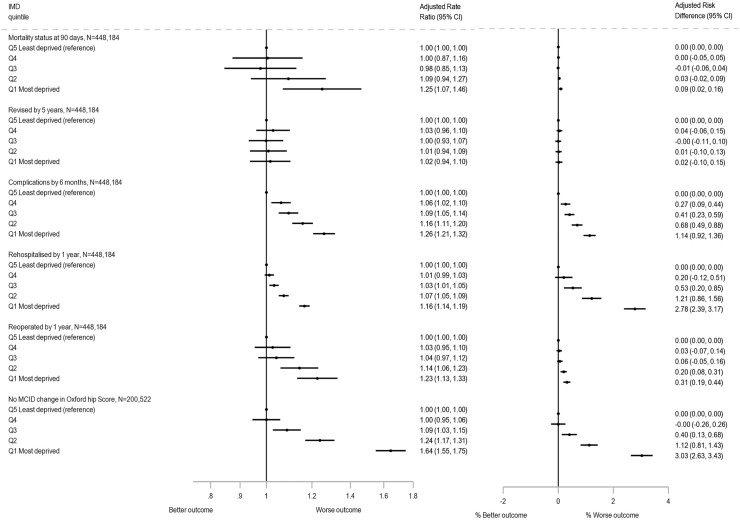
Rate ratios and risk differences for outcomes by Index of Multiple Deprivation (IMD) group adjusted for sex, age group, BMI, ASA grade and Charlson score at primary operation. MCID, Minimal Clinically Important Difference for the full Oxford Hip Score set at a five-point increase from pre- to post-total hip replacement operation (coded: 0’Improved ≥5 points’; 1’Not improved <5 points’) [21,22]. Note: All outcomes (except Oxford Hip Score) *N* = 448,184: Q5 Least deprived (reference) *n* = 106,374; Q4 *n* = 108,614; Q3 *n* = 100,676; Q2 *n* = 76,521 and Q1 Most deprived *n* = 55,999. For Oxford Hip Score *N* = 200,522: Q5 Least deprived (reference) *n* = 50,741; Q4 *n* = 50,174; Q3 *n* = 45,259; Q2 *n* = 32,612 and Q1 Most deprived *N* = 21,736. Abbreviations: ASA, American Society of Anesthesiologists’; BMI, Body Mass Index; CI, Confidence Interval; IMD, Index of Multiple Deprivation; N, number; MCID, Minimal Clinically Important Difference; Q, quintile.

Of 1,695 (0.4%) patients that died within 90 days of primary THR (S5 Table), the median time to death was 29 days, interquartile range 10–58 days. The adjusted RR of 90-day mortality reveals that there was higher mortality in the most deprived group compared to the least deprived group (Fig 2); (adjusted RR: 1.25 (95% confidence interval, CI [1.07, 1.46])). However, the absolute RD was 11 per 10,000, with an adjusted RD of 9 (95% CI [2, 16]) per 10,000 (Fig 2) and adjusted NNTH 1,105 (95% CI [640, 4,044]) (S5 Table).

### Five-year revisions

Among the 7,023 (1.6%) of patients who underwent revision surgery within the first 5 years after their primary THR (S5 Table), the median time to revision was 1.4 years, interquartile range 0.3–2.9 years. The adjusted RRs for revision, in the most deprived group compared to the least deprived group, were similar at 1.02 (95% CI [0.94, 1.10]) (Fig 2). The absolute RD was 0.12% or 12 per 10,000. The adjusted RD was 0.02% (95% CI [−0.10, 0.15]) (Fig 2) and adjusted NNTH of 4,123 with wide CIs which included number needed to benefit (NNTB) (S5 Table) [27]. Three common indications for revisions: dislocation, infection or aseptic loosening, produced similar findings to all-cause revisions.

### Complications at 6 months

**Fig 3:**
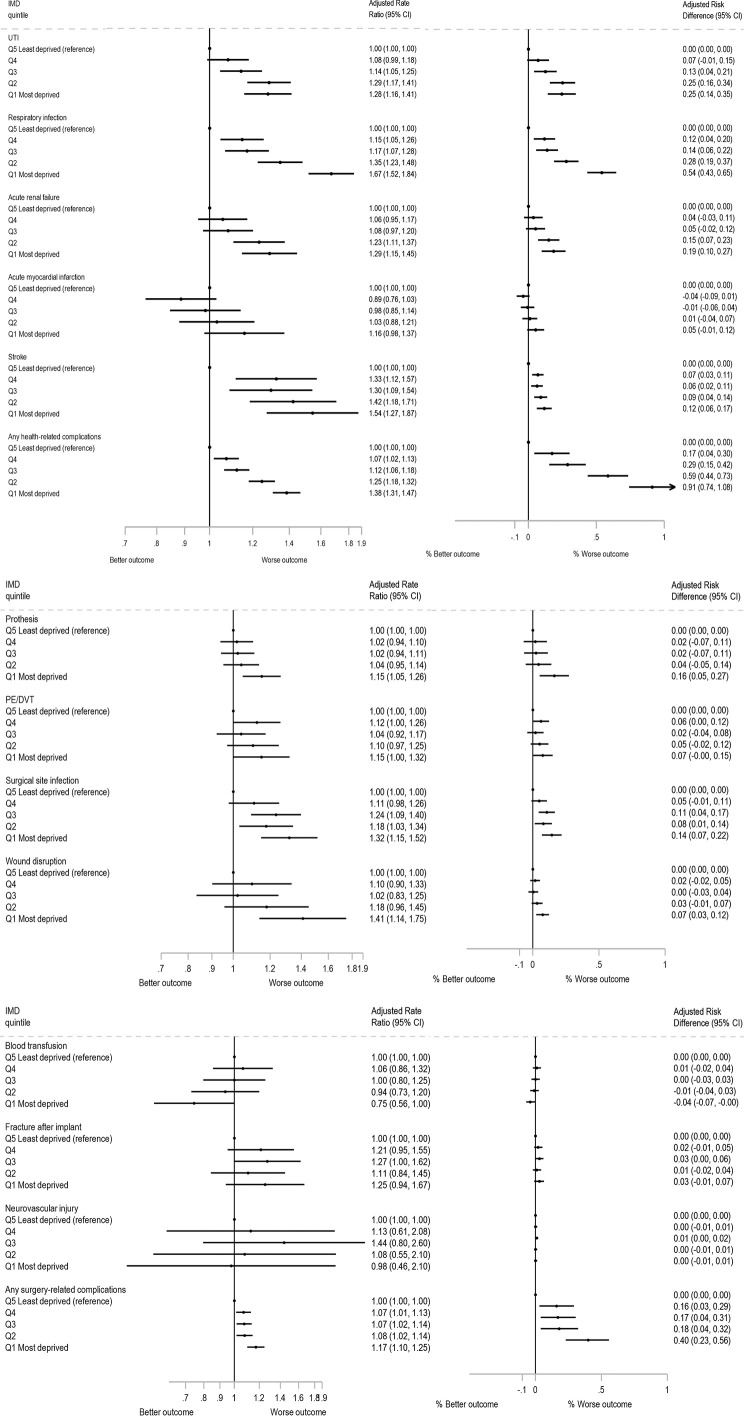
Rate ratios and risk differences for complications by Index of Multiple Deprivation (IMD) group adjusted for sex, age group, BMI, ASA grade and Charlson score at primary operation (*N* = 448,184). Note: All outcomes *N* = 448,184: Q5 Least deprived (reference) *n* = 106,374; Q4 *n* = 108,614; Q3 *n* = 100,676; Q2 *n* = 76,521 and Q1 Most deprived *n* = 55,999. Abbreviations: ASA, American Society of Anesthesiologists; BMI, Body Mass Index; CI, Confidence Interval; IMD, Index of Multiple Deprivation; N, number; PE/DVT, pulmonary embolism/deep vein thrombosis; UTI, urinary tract infection; Q, quintile.

Of 21,527 (4.8%) patients that had one or more complications within 6 months of primary THR, with 4.3% having complications in the least deprived group compared to 5.7% in the most deprived (S5 Table). Complications were more likely among those from the most deprived group compared to the least deprived group (the reference) (adjusted RR: 1.26 (95% CI [1.21, 1.32])) (Fig 2). The most common complications overall were complication of prosthesis, urinary tract infections and respiratory infections with rates of 111, 100 and 98 per 10,000 patients, respectively (S6 Table). The difference in rates of complication between the most and least deprived group were greatest for respiratory infection by 66 (95% CI [55, 78]) per 10,000 patients (adjusted RR 1.67 (95% CI [1.52, 1.84]); adjusted RD 54 (95% CI [43, 65]) per 10,000 patients and adjusted NNTH 185 (95% CI [155, 231])). In contrast, the differences were smaller for surgical related complications with prosthesis related complications being 20 (95% CI [9, 31]) per 10,000 more for the most deprived group (adjusted RR 1.15 (95% CI [1.05, 1.26]); adjusted RD 16 (95% CI [5, 27]) per 10,000 patients and adjusted NNTH 615 (95% CI [366, 1,936])) (S6 Table and Fig 3).

### Rehospitalisation and reoperation

Of 448,184 patients with linked HES data, 78,968 (17.6%) were rehospitalised for orthopaedic indications within 1 year of primary THR and 6,637 (1.5%) underwent non-revision reoperations for orthopaedic indications within 1 year of primary THR. Those from the most deprived group were more likely to be rehospitalised (adjusted RR: 1.16 (95% CI [1.14, 1.19])) or reoperated (adjusted RR: 1.23 (95% CI [1.13, 1.33])) in the year following the primary THR compared to the least deprived group (Fig 2). These findings in terms of RD were 2.78% (95% CI [2.39, 3.17]) for rehospitalisation and 0.31% (95% CI [0.19, 0.44]) for reoperation (Fig 2), with adjusted NNTH of 36 (95% CI [31, 42]) and 319 (95% CI [227, 539]), respectively (S5 Table).

### Patient reported Oxford Hip Score (OHS)

**Fig 4:**
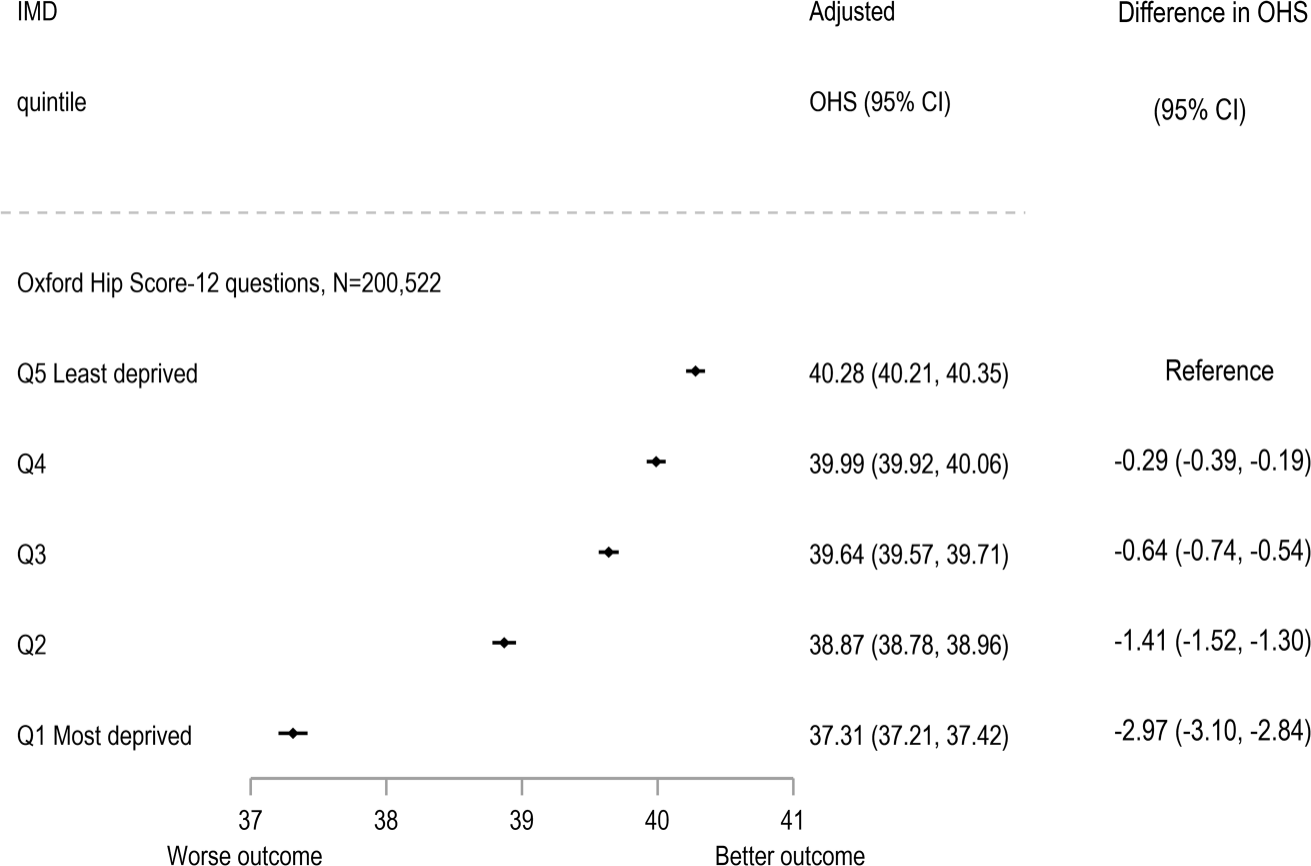
Total Oxford Hip Score (OHS) post-operation adjusted for OHS pre-operation, sex, age group, BMI, ASA grade and Charlson score by Index of Multiple Deprivation (IMD) group (*N* = 200,522). Note: For Oxford Hip Score: Q5 Least deprived (reference) *n* = 50,741; Q4 *n* = 50,174; Q3 *n* = 45,259; Q2 *n* = 32,612 and Q1 Most deprived *N* = 21,736. Abbreviations: ASA, American Society of Anesthesiologists; BMI, Body Mass Index; CI, Confidence Interval; IMD, Index of Multiple Deprivation; N, number; OHS, Oxford Hip Score; Q, quintile.

Of the 200,522 patients (45%) with valid OHS data in terms of completeness and within the time limits, the pre-operative OHS were collected at a median of 18 (interquartile range, IQR 8–37) days prior to THR surgery and post-operative scores were collected at 6.7 (IQR 6.5 to 7.4) months. Table 1 describes those with complete PROMs data in comparison to those with HES-linked data but missing valid PROMs data. The percentage missing scores in each IMD group varied from 52% to 61% (*p* < 0.001) compared to the 448,184 patients with other outcome data. There is an improvement for (unadjusted) mean pre- to mean post-operative OHS scores with similar improvements for all IMD groups (S1 Fig). Those from the most deprived group had a mean OHS up to 12 months after primary THR of 37.31 (95% CI [37.21, 37.42]) compared to those from the least deprived group (40.28 (95% CI [40.21, 40.35])) adjusted for patient characteristics and pre-operative OHS (Fig 4). The difference of −2.97 (95% CI [−3.10, −2.84]) is lower than the MCID of more than 5 points. Similar findings was seen for the subscales of pain and function (S2 Fig).

In terms of MCID, as a binary outcome change in score before and after surgery, those from the most deprived group were more likely to have worse outcome of less than five points improvement RR: 1.64 (95% CI [1.55, 1.75]) compared to the reference group of the least deprived group (Fig 2). The most deprived were therefore more likely to not improve (have <5 point difference) than the least deprived.

### Sensitivity analysis

(1) Outcomes of 90-day mortality and 5-year revision rates were examined among 550,910 patients, which included those that did not link to HES data but for whom mortality and revision NJR data were available. For these outcomes, the findings were similar: adjusted RR (most compared to least deprived): 1.34 (95% CI [1.15, 1.55]) and 1.01 (95% CI [0.94, 1.09]), respectively. Adjustment was for the same patient characteristics as the main models apart from Charlson score, as this is derived from HES data. (2a) The main outcomes of 90-day mortality, 5-year revision rates, 6-month complications and reoperation and rehospitalisation at 1 year were additionally adjusted for pre-THR OHS where available, this reduced the sample size to *N* = 260,427 and findings were broadly similar albeit with weaker associations for mortality at 90-days. (2b) As the number of operations increased over the decade, year of operation was included in the adjusted model (S3 Fig) the findings were not substantively different from those presented above.

## Discussion

Among the included patients, the number of primary first unilateral THR procedures almost doubled between 2007 and 2017, with nearly twice as many patients having THR in the least deprived group (24%) compared to the most deprived group (13%). Whilst 90-day mortality rates were low (38 per 10,000), those from the most deprived group were more likely to die within 90 days of the operation compared to the least deprived group (RR: 1.25 (95% CI [1.07, 1.46])). However, this would also be true if they had not had an operative procedure, albeit at lower rates so in terms of RD this was 9 per 10,000 (95% CI [2, 16]) with an adjusted NNTH of 1,105 (95% CI [640, 4,044]). There were similar findings for reoperations for orthopaedic indications within 1 year. Similarly, those from the most deprived compared to the least deprived group were more likely to experience health complications at 6 months; be rehospitalised for orthopaedic indications within 1 year; and report somewhat poorer pain and function outcomes although from different pre-operative states. For 5-year revisions, no variation with social deprivation was found.

This is a large national linked dataset of patients receiving primary THR surgery over 11 years. Primary procedures occurred 2007–2017 with follow-up to 2023, allowing the examination of outcomes up to 5 years following the primary procedure for the whole cohort. The chosen period means that the primary operations occurred prior to the COVID-19 pandemic and the associated service interruptions (although there is the possibility that revision rates in this period may have been affected, particularly for indications considered elective rather than urgent) [28].

Another strength of the study is that the NJR has mandatory capture of data and includes all NHS funded THRs whether performed in an NHS hospital or independent facility (estimated at 96% compliance for primary hip procedures in 2016/17) [18], so findings are more likely to be generalisable. Linkage to HES data allowed examination of further outcomes and adjustment for patient comorbidities. Although the final cohort does not include patients recorded as independently funded with THR operations undertaken in an independent hospital (e.g., who self-funded their operations or were funded by private health insurance) [18]; these patients’ data could not be linked to HES and PROMs datasets for their index procedure. It might be expected that these patients would be disproportionately likely to come from the least deprived groups, suggesting that the inequalities observed may be greater than those described. However, the sensitivity analysis which included those who self-funded or with private insurance, had similar findings for two outcomes, 90-day mortality and 5-year revisions.

Joint replacement is an elective surgery where patients typically go through a pre-operative screening or optimisation process, so there may be a healthy patient selection effect. There is evidence that those in the most deprived groups tend to have greater numbers of comorbidities [16] and be at a later stage of the disease progression prior to joint replacement surgery [29]. So that those in the most deprived groups may not be selected for surgery or selected later. The complications examined were thought to be attributable or related to having THR surgery. These complications may occur at higher rates in the deprived general population, although this could not be examined in this cohort, it would be an interesting question for further research. Data were incomplete for BMI and not available for patient smoking status; these are factors that should be considered when examining differences in outcomes between patient groups.

For patients who underwent bilateral THRs on different dates, only the first procedure was included in the analysis. Laterality was known for these initial procedures, allowing PROMs, subsequent revisions—and, in most cases, reoperations—to be matched to the correct hip. Although some recorded rehospitalisations may have been related to the second hip replacement, the number of such cases is expected to be small. An alternative approach would have been to exclude all cases involving bilateral procedures on different dates; however, inclusion was preferred to increase the generalisability of findings.

There were limited PROMs data, with data available for less than 50% of patients in the cohort [30] hence these findings should be interpreted with caution. PROMs data have only been extensively collected in more recent years, although those from the most deprived groups are disproportionately less likely to be included [9]. In common with the other outcomes, patients needed to survive to the measured time point, in order to be able to contribute PROMs data. There are known differences in mortality and revision rates for patients with complete PROMs data compared to those with missing data [18].

Few studies report area-level inequalities for a wide range of outcomes following THR, including patient reported measures, in a national cohort using linked data. Of those studies that report area-level inequalities findings are inconsistent. Similar to this study other studies of varying sizes, using a variety of area-level deprivation measures, have reported that for THR those from the most deprived areas had increased risk of 90-day mortality [8,9]; whilst others reported no association with 30-day [8,10], 90-day [12], and total mortality [11,12]. For risk of THR revision, several studies in agreement with this study, reported there was no association with area-level deprivation at various time points, at or after 4 [11] or 5 years [13].

Post-operative complications measured in varying ways, were reported as having no or weak association with deprivation at 90 days [11], and to 1 year [8,14], with similar findings for specific complications: infection, thromboembolism and dislocation [12]. In contrast, one study did report a strong association with dislocation [8,9,15]. No studies examined rehospitalisation or reoperation specifically for an orthopaedic indication up to 1 year, which were examined in this study, although all-cause 30- and 90-day readmission has been examined with some studies finding no association [8,31].

Whilst a systematic review of total joint arthroplasty and PROMs had mixed findings [8], two included studies found relative to pre-operative scores, OHS improved for all groups, and generally the least deprived patients had a greater improvement than the most deprived patients [9,16]. In the present study, even though those from the most deprived groups reported lower (worse) OHS before and after surgery, there was a substantial improvement in OHS in all socioeconomic groups.

Patients living in deprived areas tend to be at a later stage of disease progression before they have THR surgery [16], and this may influence outcomes for this group. Examining mortality at 90 days allows examination of death in the period proximal to the insult of the primary operation, and so mortality may be more likely to be attributable to the THR operation, rather than comorbidities or an interaction between the surgical procedure and pre-existing morbidities [32]. This may explain why the RDs for surgery-related complications were relatively small. The largest difference was observed for respiratory infections. In this case it is likely that operative procedures may have triggered pre-existing socioeconomic differences in chronic obstructive airways disease. Adjustment for pre-operative ASA grade was included in all models, but will not have fully captured patient comorbidity, hence this study additionally adjusted for Charlson Score [24]. Even with this there is likely to be residual confounding and pre-operative measures of lung function would have been better still, but are not available in these data. The lack of socioeconomic differences for revision of THR at five years is interesting and has several potential reasons. There may truly be no difference in risk, but patients needed to survive and be well enough for revision surgery (“survival bias”); this may in part explain why no pattern with deprivation was observed as those needing revision may be at greater risk of death. There may also be clinician/patient-driven thresholds for revision surgery applied differently between socioeconomic groups, as evidenced by lower provision of THR relative to need in more deprived groups [7].

This study provides evidence of socioeconomic inequalities in complications and rehospitalisations, with those from deprived areas having worse outcomes, although these differences particularly in terms of RDs were weaker for short-term mortality and reoperations, and not seen for 5-year revision rates. The size of these differences in both relative and absolute terms may be interpreted differently by patients and clinicians depending on their degree of risk aversion. However, all groups gained equally in terms of PROMs albeit from different pre-operative states. These findings are useful measures to inform shared decision-making for patients choosing whether to undergo hip replacement, and to benchmark the effectiveness of policies to reduce inequalities in the care pathways for the provision of THR [1,4,5].

In England, inequalities in several outcomes following THR are present by area-level deprivation. These findings can inform shared decision-making between patients and clinicians when deciding whether to undergo hip replacement, and serve as benchmarks for evaluating policies aimed at reducing health inequalities after THR.

## Supporting information

S1 Checklist The RECORD statement—checklist of items, extended from the STROBE statement, that should be reported in observational studies using routinely collected health data. (PDF)

S1 Fig Unadjusted Oxford Hip Score (OHS) mean pre-operative score prior to total hip replacement (95%CI) versus mean post-operative score (95%CI) by Index of Multiple Deprivation (IMD) group (*N* = 200,522). (DOCX)

S2 Fig Oxford Hip Score (OHS) subscales for pain and function post-operation adjusted for OHS pre-operation score, sex, age group, BMI, ASA grade and Charlson score by Index of Multiple Deprivation (IMD) group. (DOCX)

S3 Fig Rate ratios for outcomes by Index of Multiple Deprivation (IMD) group adjusted for sex, age group, BMI, ASA grade and Charlson score at primary operation, with the addition of year of surgery. (DOCX)

S1 Table International Classification of Diseases, and Classification of Interventions and Procedures, OPCS-4 codes used to identify complications. (DOCX)

S2 Table International Classification of Diseases, ICD10 codes used to identify rehospitalisations for orthopaedic indications. (DOCX)

S3 Table Classification of Interventions and Procedures, OPCS-4 codes used to identify reoperations for orthopaedic indications. (DOCX)

S4 Table Characteristics of 448,184 patients at primary hip replacement in England by Index of Multiple Deprivation (IMD) (2007–2017). (DOCX)

S5 Table Outcomes for patients following primary hip replacement in England for patients with linked HES or PROMs data by Index of Multiple Deprivation (IMD) (2007–2017). (DOCX)

S6 Table Cumulative complications at 6 months post hip replacement by Index of Multiple Deprivation (IMD) group (*N* = 448,184). (DOCX)

## Abbreviations

ASA: American Society of Anesthesiologists
BMI: body mass index
CI: confidence interval
HES: Hospital Episode Statistics
ICD: International Classification of Diseases
IMD: Index of Multiple Deprivation
IQR: interquartile range
LSOAs: Lower Layer Super Output Areas
MCID: Minimal Clinically Important Difference
NHS: National Health Service
NJR: National Joint Registry
NNTH: number needed to harm
OHS: Oxford Hip Score
PROM: Patient Reported Outcome Measure
RD: risk difference
RR: rate ratio
THR: total hip replacement
