# Supplementary material for: Social inequalities in patient outcomes after total hip replacement surgery for osteoarthritis in England: A population-based cohort study of the National Joint Registry
Source: PLoS Med. 2026 Feb 2;23(2):e1004870. doi: 10.1371/journal.pmed.1004870 (PMC12863669; doi:10.1371/journal.pmed.1004870)
Supplement: S1 Fig — (DOCX) [file pmed.1004870.s002.docx]

S1 Fig: Unadjusted Oxford Hip Score (OHS) mean pre-operative score prior to total hip replacement (95%CI) versus mean post-operative score (95%CI) by Index of Multiple Deprivation (IMD) group (N=200,522)

Abbreviations: CI, Confidence Interval; IMD, Index of Multiple Deprivation; OHS, Oxford Hip Score; Q, quintile
