# Supplementary material for: Social inequalities in patient outcomes after total hip replacement surgery for osteoarthritis in England: A population-based cohort study of the National Joint Registry
Source: PLoS Med. 2026 Feb 2;23(2):e1004870. doi: 10.1371/journal.pmed.1004870 (PMC12863669; doi:10.1371/journal.pmed.1004870)
Supplement: S2 Fig — (DOCX) [file pmed.1004870.s003.docx]

S2 Fig: Oxford Hip Score (OHS) subscales for pain and function post-operation adjusted for OHS pre-operation score, sex, age group, BMI, ASA grade and Charlson score by Index of Multiple Deprivation (IMD) group

Difference in OHS

(95% CI)

Reference

-0.14 (-0.19, -0.08)

-0.35 (-0.40, -0.29)

-0.74 (-0.80, -0.67)

-1.56 (-1.63, -1.48)

Reference

-0.16 (-0.21, -0.11)

-0.30 (-0.35, -0.25)

-0.68 (-0.74, -0.62)

-1.42 (-1.48, -1.35)

Abbreviations: ASA, American Society of Anesthesiologists; BMI, Body Mass Index; CI, Confidence Interval; IMD, Index of Multiple Deprivation; N, number; OHS, Oxford Hip Score; Q, quintile
