# Supplementary material for: Social inequalities in patient outcomes after total hip replacement surgery for osteoarthritis in England: A population-based cohort study of the National Joint Registry
Source: PLoS Med. 2026 Feb 2;23(2):e1004870. doi: 10.1371/journal.pmed.1004870 (PMC12863669; doi:10.1371/journal.pmed.1004870)
Supplement: S3 Fig — (DOCX) [file pmed.1004870.s004.docx]

S3 Fig: Rate ratios for outcomes by Index of Multiple Deprivation (IMD) group adjusted for sex, age group, BMI, ASA grade and Charlson score at primary operation, with the addition of year of surgery

MCID, Minimal Clinically Important Difference for the full Oxford Hip Score set at a five-point increase from pre- to post-total hip replacement operation (coded: 0 ’Improved >=5 points’; 1 ’Not improved <5 points’)

Abbreviations: ASA, American Society of Anesthesiologists’; BMI, Body Mass Index; CI, Confidence Interval; IMD, Index of Multiple Deprivation; N, number; MCID, Minimal Clinically Important Difference; Q, quintile
