# Supplementary material for: Social inequalities in patient outcomes after total hip replacement surgery for osteoarthritis in England: A population-based cohort study of the National Joint Registry
Source: PLoS Med. 2026 Feb 2;23(2):e1004870. doi: 10.1371/journal.pmed.1004870 (PMC12863669; doi:10.1371/journal.pmed.1004870)
Supplement: S1 Table — (DOCX) [file pmed.1004870.s005.docx]

S4 Table: Characteristics of 448,184 patients at primary hip replacement in England by Index of Multiple Deprivation (IMD) (2007 to 2017)

|  |  |  | |  |  | |  | | IMD | |  | |  | |  |
| --- | --- | --- | --- | --- | --- | --- | --- | --- | --- | --- | --- | --- | --- | --- | --- |
|  |  | **Total** | |  | **Q5 Least deprived** | | **Q4** | | **Q3** | | **Q2** | | **Q1 Most deprived** | | **Chi2** |
|  |  | **N** | **%** |  | **N** | **%** | **N** | **%** | **N** | **%** | **N** | **%** | **N** | **%** |  |
| Total | N | 448,184 | 100 |  | 106,374 | 23.7 | 108,614 | 24.2 | 100,676 | 22.5 | 76,521 | 17.1 | 55,999 | 12.5 |  |
| Sex | Female | 272,974 | 60.9 |  | 64,817 | 60.9 | 65,374 | 60.2 | 60,969 | 60.6 | 46,944 | 61.3 | 34,870 | 62.3 |  |
|  | Male | 175,210 | 39.1 |  | 41,557 | 39.1 | 43,240 | 39.8 | 39,707 | 39.4 | 29,577 | 38.7 | 21,129 | 37.7 | p<0.001 |
| Age at primary (years) | 50 to 54 | 22,737 | 5.1 |  | 4,478 | 4.2 | 5,007 | 4.6 | 4,942 | 4.9 | 4,436 | 5.8 | 3,874 | 6.9 |  |
|  | 55 to 64 | 96,418 | 21.5 |  | 20,724 | 19.5 | 22,389 | 20.6 | 21,715 | 21.6 | 17,356 | 22.7 | 14,234 | 25.4 |  |
|  | 65 to 74 | 173,200 | 38.6 |  | 42,081 | 39.6 | 42,620 | 39.2 | 39,173 | 38.9 | 28,912 | 37.8 | 20,414 | 36.5 |  |
|  | 75+ | 155,829 | 34.8 |  | 39,091 | 36.8 | 38,598 | 35.5 | 34,846 | 34.6 | 25,817 | 33.7 | 17,477 | 31.2 | p<0.001 |
| Body Mass Index at primary (kg/m^2^) | <18.5 | 2,471 | 0.6 |  | 621 | 0.6 | 592 | 0.6 | 542 | 0.5 | 389 | 0.5 | 327 | 0.6 |  |
|  | 18.5 to 24.9 | 59,091 | 13.2 |  | 15,864 | 14.9 | 15,097 | 13.9 | 12,911 | 12.8 | 9,070 | 11.9 | 6,149 | 11 |  |
|  | 25 to 29.9 | 122,466 | 27.3 |  | 31,265 | 29.4 | 30,784 | 28.3 | 27,318 | 27.1 | 19,878 | 26 | 13,221 | 23.6 |  |
|  | >29.9 | 122,911 | 27.4 |  | 26,141 | 24.6 | 28,656 | 26.4 | 28,065 | 27.9 | 22,551 | 29.5 | 17,498 | 31.2 |  |
|  | Unknown BMI | 141,245 | 31.5 |  | 32,483 | 30.5 | 33,485 | 30.8 | 31,840 | 31.6 | 24,633 | 32.2 | 18,804 | 33.6 | p<0.001 |
| ASA grade | P1 - Fit and healthy | 51,890 | 11.6 |  | 13,908 | 13.1 | 13,401 | 12.3 | 11,691 | 11.6 | 8,090 | 10.6 | 4,800 | 8.6 |  |
|  | P2 - Mild disease not incapacitating | 319,193 | 71.2 |  | 76,768 | 72.2 | 77,577 | 71.4 | 71,888 | 71.4 | 53,955 | 70.5 | 39,005 | 69.7 |  |
|  | P3 - Incapacitating systemic disease | 74,923 | 16.7 |  | 15,283 | 14.4 | 17,173 | 15.8 | 16,592 | 16.5 | 14,056 | 18.4 | 11,819 | 21.1 |  |
|  | P4 - Life threatening disease/P5 - Expected to die within 24hrs | 2,178 | 0.5 |  | 415 | 0.4 | 463 | 0.4 | 505 | 0.5 | 420 | 0.6 | 375 | 0.7 | p<0.001 |
| Charlson score (morbidity) | None | 289,326 | 64.6 |  | 71,553 | 67.3 | 71,797 | 66.1 | 65,516 | 65.1 | 47,750 | 62.4 | 32,710 | 58.4 |  |
|  | Mild | 82,786 | 18.5 |  | 17,695 | 16.6 | 18,783 | 17.3 | 18,409 | 18.3 | 15,202 | 19.9 | 12,697 | 22.7 |  |
|  | Moderate | 45,235 | 10.1 |  | 10,614 | 10 | 10,886 | 10 | 9,988 | 9.9 | 7,920 | 10.4 | 5,827 | 10.4 |  |
|  | Severe | 30,837 | 6.9 |  | 6,512 | 6.1 | 7,148 | 6.6 | 6,763 | 6.7 | 5,649 | 7.4 | 4,765 | 8.5 | p<0.001 |
| Year | 2007 | 26,928 | 6 |  | 5,965 | 5.6 | 6,317 | 5.8 | 6,146 | 6.1 | 4,875 | 6.4 | 3,625 | 6.5 |  |
|  | 2008 | 29,589 | 6.6 |  | 6,554 | 6.2 | 6,940 | 6.4 | 6,755 | 6.7 | 5,363 | 7 | 3,977 | 7.1 |  |
|  | 2009 | 31,726 | 7.1 |  | 7,235 | 6.8 | 7,708 | 7.1 | 7,230 | 7.2 | 5,528 | 7.2 | 4,025 | 7.2 |  |
|  | 2010 | 37,056 | 8.3 |  | 8,702 | 8.2 | 8,945 | 8.2 | 8,344 | 8.3 | 6,352 | 8.3 | 4,713 | 8.4 |  |
|  | 2011 | 40,007 | 8.9 |  | 9,397 | 8.8 | 9,682 | 8.9 | 9,042 | 9 | 6,806 | 8.9 | 5,080 | 9.1 |  |
|  | 2012 | 43,514 | 9.7 |  | 10,405 | 9.8 | 10,434 | 9.6 | 9,811 | 9.8 | 7,437 | 9.7 | 5,427 | 9.7 |  |
|  | 2013 | 44,698 | 10 |  | 10,626 | 10 | 10,821 | 10 | 10,112 | 10 | 7,541 | 9.9 | 5,598 | 10 |  |
|  | 2014 | 48,281 | 10.8 |  | 11,860 | 11.1 | 11,632 | 10.7 | 10,806 | 10.7 | 8,131 | 10.6 | 5,852 | 10.4 |  |
|  | 2015 | 48,346 | 10.8 |  | 11,616 | 10.9 | 11,905 | 11 | 10,666 | 10.6 | 8,199 | 10.7 | 5,960 | 10.6 |  |
|  | 2016 | 49,555 | 11.1 |  | 12,234 | 11.5 | 12,292 | 11.3 | 10,887 | 10.8 | 8,254 | 10.8 | 5,888 | 10.5 |  |
|  | 2017 | 48,484 | 10.8 |  | 11,780 | 11.1 | 11,938 | 11 | 10,877 | 10.8 | 8,035 | 10.5 | 5,854 | 10.4 |  |
| Valid PROMs data available | Yes | 200,522 | 44.7 |  | 50,741 | 25.3 | 50,174 | 25.0 | 45,259 | 22.6 | 32,612 | 16.3 | 21,736 | 10.8 | p<0.001 |
|  | % of those in each IMD group that have valid PROMs data (column %) |  | 44.7 |  |  | 47.7 |  | 46.2 |  | 45.0 |  | 42.6 |  | 38.8 |  |

Abbreviations: ASA, American Society of Anesthesiologists; BMI, Body Mass Index; IMD, Index of Multiple Deprivation; PROMs Patient Reported Outcome Measures; Q, quintile
