# Supplementary material for: Social inequalities in patient outcomes after total hip replacement surgery for osteoarthritis in England: A population-based cohort study of the National Joint Registry
Source: PLoS Med. 2026 Feb 2;23(2):e1004870. doi: 10.1371/journal.pmed.1004870 (PMC12863669; doi:10.1371/journal.pmed.1004870)
Supplement: S2 Table — (DOCX) [file pmed.1004870.s006.docx]

S2 Table: International Classification of Diseases, ICD10 codes used to identify rehospitalisations for orthopaedic indications

| **ICD10 code** | **DESCRIPTION** |
| --- | --- |
| M0015 | Pneumococcal arthritis and polyarthritis |
| M0025 | Other streptococcal arthritis and polyarthritis |
| M0085 | Arthritis and polyarthritis due to other specified bacterial agents |
| M0095 | Pyogenic arthritis, unspecified |
| M0105 | Meningococcal arthritis |
| M0135 | Arthritis in other bacterial diseases classified elsewhere |
| M16 | Coxarthrosis [arthrosis of hip] |
| M160 | Primary coxarthrosis, bilateral |
| M161 | Other primary coxarthrosis |
| M162 | Coxarthrosis resulting from dysplasia, bilateral |
| M163 | Other dysplastic coxarthrosis |
| M164 | Post-traumatic coxarthrosis, bilateral |
| M165 | Other post-traumatic coxarthrosis |
| M166 | Other secondary coxarthrosis, bilateral |
| M167 | Other secondary coxarthrosis |
| M169 | Coxarthrosis, unspecified |
| M2135 | M21.35 Wrist or foot drop (acquired)-Pelvic/thigh |
| M2136 | M21.36 Wrist or foot drop (acquired)-Lower leg |
| M2137 | Wrist or foot drop (acquired) |
| M2138 | M21.38 Wrist or foot drop (acquired)-Other |
| M2175 | Unequal limb length (acquired) |
| M2405 | Loose body in joint |
| M2435 | Pathological dislocation and subluxation of joint, not elsewhere classified |
| M2445 | Recurrent dislocation and subluxation of joint |
| M2455 | Contracture of joint |
| M2465 | Ankylosis of joint |
| M247 | Protrusio acetabuli |
| M2470 | M24.70 Protrusio acetabuli-Mult sites |
| M2475 | M24.75 Protrusio acetabuli-Pelvic/thigh |
| M2478 | M24.78 Protrusio acetabuli-Other |
| M2479 | M24.79 Protrusio acetabuli-Site unspec |
| M2485 | Other specific joint derangements, not elsewhere classified |
| M2495 | Joint derangement, unspecified |
| M2505 | Haemarthrosis |
| M2515 | Fistula of joint |
| M2525 | Flail joint |
| M2535 | Other instability of joint |
| M2545 | Effusion of joint |
| M2555 | Pain in joint |
| M2565 | Stiffness of joint, not elsewhere classified |
| M2585 | Other specified joint disorders |
| M2595 | Joint disorder, unspecified |
| M6145 | Other calcification of muscle |
| M6155 | Other ossification of muscle |
| M6195 | Calcification and ossification of muscle, unspecified |
| M706 | Trochanteric bursitis |
| M7060 | M70.60 Trochanteric bursitis-Mult sites |
| M7065 | M70.65 Trochanteric bursitis-Pelvic/Thigh |
| M7068 | M70.68 Trochanteric bursitis-Other |
| M7069 | M70.69 Trochanteric bursitis-Site unspec |
| M707 | Other bursitis of hip |
| M7070 | M70.70 Other bursitis of hip-Mult sites |
| M7075 | M70.75 Other bursitis of hip-Pelvic/Thigh |
| M7078 | M70.78 Other bursitis of hip-Other |
| M7079 | M70.79 Other bursitis of hip-Site unspec |
| M7105 | Abscess of bursa |
| M7115 | Other infective bursitis |
| M7135 | Other bursal cyst |
| M7145 | Calcium deposit in bursa |
| M7155 | Other bursitis, not elsewhere classified |
| M7185 | Other specified bursopathies |
| M7195 | Bursopathy, unspecified |
| M7265 | Necrotizing fasciitis |
| M760 | Gluteal tendinitis |
| M7600 | Gluteal tendinitis |
| M7605 | Gluteal tendinitis |
| M7608 | M76.08 Gluteal tendinitis-Other |
| M7609 | M76.09 Gluteal tendinitis-Site unspec |
| M761 | Psoas tendinitis |
| M7610 | Psoas tendinitis |
| M7615 | Psoas tendinitis |
| M7618 | M76.18 Psoas tendinitis-Other |
| M7619 | M76.19 Psoas tendinitis-Site unspec |
| M763 | Iliotibial band syndrome |
| M7630 | Iliotibial band syndrome |
| M7635 | Iliotibial band syndrome |
| M7638 | M76.38 Iliotibial band syndrome-Other |
| M7639 | M76.39 Iliotibial band syndrome-Site unspec |
| M7695 | Enthesopathy of lower limb, unspecified |
| M7785 | Other enthesopathies, not elsewhere classified |
| M7795 | Enthesopathy, unspecified |
| M7955 | Residual foreign body in soft tissue |
| M7965 | Pain in limb |
| M8585 | Other specified disorders of bone density and structure |
| M8595 | Disorder of bone density and structure, unspecified |
| M8605 | Acute haematogenous osteomyelitis |
| M8615 | Other acute osteomyelitis |
| M8625 | Subacute osteomyelitis |
| M8635 | Chronic multifocal osteomyelitis |
| M8645 | Chronic osteomyelitis with draining sinus |
| M8655 | Other chronic haematogenous osteomyelitis |
| M8665 | Other chronic osteomyelitis |
| M8685 | Other osteomyelitis |
| M8695 | Osteomyelitis, unspecified |
| M8705 | Idiopathic aseptic necrosis of bone |
| M8935 | Hypertrophy of bone |
| M8945 | Other hypertrophic osteoarthropathy |
| M8955 | Osteolysis |
| M8985 | Other specified disorders of bone |
| M8995 | Disorder of bone, unspecified |
| M9005 | Tuberculosis of bone |
| M9015 | Periostitis in other infectious diseases classified elsewhere |
| M96 | Postprocedural musculoskeletal disorders, not elsewhere classified |
| M966 | Fracture of bone following insertion of orthopaedic implant, joint prosthesis, or bone plate |
| M9665 | M96.65 Fracture of pelvis following insertion of orthopedic implant, joint prosthesis, or bone plate (Fx pelvis following insrt ortho implnt/prosth/bone plt) |
| M968 | Other postprocedural musculoskeletal disorders |
| M969 | Postprocedural musculoskeletal disorder, unspecified |
| Q656 | Unstable hip |
| S72 | Fracture of femur |
| S720 | Fracture of neck of femur |
| S7200 | Fracture of neck of femur |
| S7201 | Fracture of neck of femur |
| S721 | Pertrochanteric fracture |
| S7210 | Pertrochanteric fracture |
| S7211 | Pertrochanteric fracture |
| S722 | Subtrochanteric fracture |
| S7220 | Subtrochanteric fracture |
| S7221 | Subtrochanteric fracture |
| S723 | Fracture of shaft of femur |
| S7230 | Fracture of shaft of femur |
| S7231 | Fracture of shaft of femur |
| S724 | Fracture of lower end of femur |
| S7240 | Fracture of lower end of femur |
| S7241 | Fracture of lower end of femur |
| S727 | Multiple fractures of femur |
| S7270 | Multiple fractures of femur |
| S7271 | Multiple fractures of femur |
| S728 | Fractures of other parts of femur |
| S7280 | Fractures of other parts of femur |
| S7281 | Fractures of other parts of femur |
| S729 | Fracture of femur, part unspecified |
| S7290 | Fracture of femur, part unspecified |
| S7291 | Fracture of femur, part unspecified |
| S73 | Dislocation, sprain and strain of joint and ligaments of hip |
| S730 | Dislocation of hip |
| S731 | Sprain and strain of hip |
| S74 | Injury of nerves at hip and thigh level |
| S740 | Injury of sciatic nerve at hip and thigh level |
| S741 | Injury of femoral nerve at hip and thigh level |
| S742 | Injury of cutaneous sensory nerve at hip and thigh level |
| S747 | Injury of multiple nerves at hip and thigh level |
| S748 | Injury of other nerves at hip and thigh level |
| S749 | Injury of unspecified nerve at hip and thigh level |
| S75 | Injury of blood vessels at hip and thigh level |
| S750 | Injury of femoral artery |
| S751 | Injury of femoral vein at hip and thigh level |
| S752 | Injury of greater saphenous vein at hip and thigh level |
| S757 | Injury of multiple blood vessels at hip and thigh level |
| S758 | Injury of other blood vessels at hip and thigh level |
| S759 | Injury of unspecified blood vessel at hip and thigh level |
| T81 | Complications of procedures, not elsewhere classified |
| T810 | Haemorrhage and haematoma complicating a procedure, not elsewhere classified |
| T811 | Shock during or resulting from a procedure, not elsewhere classified |
| T812 | Accidental puncture and laceration during a procedure, not elsewhere classified |
| T813 | Disruption of operation wound, not elsewhere classified |
| T814 | Infection following a procedure, not elsewhere classified |
| T815 | Foreign body accidentally left in body cavity or operation wound following a procedure |
| T816 | Acute reaction to foreign substance accidentally left during a procedure |
| T817 | Vascular complications following a procedure, not elsewhere classified |
| T818 | Other complications of procedures, not elsewhere classified |
| T819 | Unspecified complication of procedure |
| T84 | Complications of internal orthopaedic prosthetic devices, implants and grafts |
| T840 | Mechanical complication of internal joint prosthesis |
| T841 | Mechanical complication of internal fixation device of bones of limb |
| T843 | Mechanical complication of other bone devices, implants and grafts |
| T844 | Mechanical complication of other internal orthopaedic devices, implants and grafts |
| T845 | Infection and inflammatory reaction due to internal joint prosthesis |
| T846 | Infection and inflammatory reaction due to internal fixation device [any site] |
| T847 | Infection and inflammatory reaction due to other internal orthopaedic prosthetic devices, implants and grafts |
| T848 | Other complications of internal orthopaedic prosthetic devices, implants and grafts |
| T849 | Unspecified complication of internal orthopaedic prosthetic device, implant and graft |
| T85 | Complications of other internal prosthetic devices, implants and grafts |
| T856 | Mechanical complication of other specified internal prosthetic devices, implants and grafts |
| T857 | Infection and inflammatory reaction due to other internal prosthetic devices, implants and grafts |
| T858 | Other complications of internal prosthetic devices, implants and grafts, not elsewhere classified |
| T859 | Unspecified complication of internal prosthetic device, implant and graft |
| T931 | Sequelae of fracture of femur |
| T933 | Sequelae of dislocation, sprain and strain of lower limb |
| Y79 | Orthopaedic devices associated with adverse incidents |
| Y792 | Orthopaedic devices associated with adverse incidents |
| Y793 | Orthopaedic devices associated with adverse incidents |
| Y798 | Orthopaedic devices associated with adverse incidents |
| Y83 | Surgical operation and other surgical procedures as the cause of abnormal reaction of the patient, or of later complication, without mention of misadventure at the time of the procedure |
| Y831 | Surgical operation with implant of artificial internal device |
| Z467 | Fitting and adjustment of orthopaedic device |
| Z47 | Other orthopaedic follow-up care |
| Z470 | Follow-up care involving removal of fracture plate and other internal fixation device |
| Z478 | Other specified orthopaedic follow-up care |
| Z479 | Orthopaedic follow-up care, unspecified |
| Z966 | Presence of orthopaedic joint implants |
