# Supplementary material for: Social inequalities in patient outcomes after total hip replacement surgery for osteoarthritis in England: A population-based cohort study of the National Joint Registry
Source: PLoS Med. 2026 Feb 2;23(2):e1004870. doi: 10.1371/journal.pmed.1004870 (PMC12863669; doi:10.1371/journal.pmed.1004870)
Supplement: S3 Table — (DOCX) [file pmed.1004870.s007.docx]

S3 Table: Classification of Interventions and Procedures, OPCS-4 codes used to identify reoperations for orthopaedic indications

Location code (Z843 Hip joint, Z756 Acetabulum, Z761 Head of femur, Z902 Hip NEC) and laterality (Z943 left, Z942 right, Z94.1 bilateral) were also used to identify reoperations for orthopaedic indications

| **OPCS-4 code** | **Description** |
| --- | --- |
| O198 | Other specified other therapeutic endoscopic operations on other joint structure |
| O199 | Unspecified other therapeutic endoscopic operations on other joint structure |
| O271 | Extra-articular ligament reconstruction for stabilisation of joint |
| O278 | Other specified other stabilising operations on joint |
| O279 | Unspecified other stabilising operations on joint |
| O298 | Other specified excision of bone |
| O299 | Unspecified excision of bone |
| S172 | Distant myocutaneous subcutaneous pedicle flap NEC |
| S175 | Distant myocutaneous free flap NEC |
| S178 | Other specified distant flap of skin and muscle |
| S179 | Unspecified distant flap of skin and muscle |
| S182 | Distant fasciocutaneous subcutaneous pedicle flap NEC |
| S185 | Distant fasciocutaneous free flap NEC |
| S188 | Other specified distant flap of skin and fascia |
| S189 | Unspecified distant flap of skin and fascia |
| S192 | Distant tube pedicle flap of skin NEC |
| S198 | Other specified distant pedicle flap of skin |
| S199 | Unspecified distant pedicle flap of skin |
| S202 | Axial pattern distant flap of skin NEC |
| S204 | Random pattern distant flap of skin NEC |
| S207 | Distant free flap of skin NEC |
| S208 | Other specified other distant flap of skin |
| S209 | Unspecified other distant flap of skin |
| S232 | Z plasty NEC |
| S234 | W plasty NEC |
| S238 | Other specified flap operations to relax contracture of skin |
| S239 | Unspecified flap operations to relax contracture of skin |
| S242 | Local myocutaneous subcutaneous pedicle flap NEC |
| S248 | Other specified local flap of skin and muscle |
| S249 | Unspecified local flap of skin and muscle |
| S252 | Local fasciocutaneous subcutaneous pedicle flap NEC |
| S258 | Other specified local flap of skin and fascia |
| S259 | Unspecified local flap of skin and fascia |
| S262 | Axial pattern local subcutaneous pedicle flap of skin NEC |
| S264 | Random pattern local subcutaneous pedicle flap of skin NEC |
| S268 | Other specified local subcutaneous pedicle flap of skin |
| S269 | Unspecified local subcutaneous pedicle flap of skin |
| S272 | Axial pattern local flap of skin NEC |
| S274 | Random pattern local flap of skin NEC |
| S278 | Other specified other local flap of skin |
| S279 | Unspecified other local flap of skin |
| S31 | Other operations on flap of skin to other site |
| S311 | Delay of flap of skin NEC |
| S312 | Transfer of flap of skin NEC |
| S313 | Revision of flap of skin NEC |
| S314 | Final inset of flap of skin NEC |
| S315 | Thinning of flap of skin NEC |
| S316 | Removal of flap of skin NEC |
| S318 | Other specified other operations on flap of skin to other site |
| S319 | Unspecified other operations on flap of skin to other site |
| S352 | Meshed split autograft of skin NEC |
| S358 | Other specified split autograft of skin |
| S359 | Unspecified split autograft of skin |
| S362 | Full thickness autograft of skin NEC |
| S364 | Composite autograft of skin NEC |
| S366 | Pinch graft of skin NEC |
| S368 | Other specified other autograft of skin |
| S369 | Unspecified other autograft of skin |
| S372 | Allograft of skin NEC |
| S374 | Xenograft of skin NEC |
| S378 | Other specified other graft of skin |
| S379 | Unspecified other graft of skin |
| S398 | Other specified graft of other tissue to skin |
| S399 | Unspecified graft of other tissue to skin |
| S401 | Tape closure of skin NEC |
| S402 | Tissue adhesive closure of skin NEC |
| S408 | Other specified other closure of skin |
| S409 | Unspecified other closure of skin |
| S476 | Incision of skin NEC |
| S478 | Other specified opening of skin |
| S479 | Unspecified opening of skin |
| S57 | Exploration of other skin of other site |
| S571 | Debridement of skin NEC |
| S572 | Removal of slough from skin NEC |
| S573 | Toilet of skin NEC |
| S574 | Dressing of skin NEC |
| S575 | Attention to dressing of skin NEC |
| S576 | Cleansing and sterilisation of skin NEC |
| S577 | Dressing of skin using vacuum assisted closure device NEC |
| S578 | Other specified exploration of other skin of other site |
| S579 | Unspecified exploration of other skin of other site |
| S588 | Other specified larvae therapy of skin |
| S589 | Unspecified larvae therapy of skin |
| S604 | Refashioning of scar NEC |
| S624 | Removal of pack from subcutaneous tissue |
| S628 | Other specified other operations on subcutaneous tissue |
| S629 | Unspecified other operations on subcutaneous tissue |
| T342 | Open drainage of pelvic abscess |
| T452 | Image controlled percutaneous drainage of pelvic abscess |
| T62 | Operations on bursa |
| T621 | Total excision of bursa |
| T622 | Excision of bursa NEC |
| T623 | Biopsy of lesion of bursa |
| T624 | Aspiration of bursa |
| T625 | Injection into bursa |
| T626 | Exploration of bursa |
| T628 | Other specified operations on bursa |
| T629 | Unspecified operations on bursa |
| T64 | Transposition of tendon |
| T641 | Multiple transfer of tendon to tendon |
| T642 | Transfer of tendon to tendon NEC |
| T643 | Multiple insertion of tendons into bone |
| T644 | Insertion of tendon into bone NEC |
| T645 | Tenodesis |
| T648 | Other specified transposition of tendon |
| T649 | Unspecified transposition of tendon |
| T65 | Excision of tendon |
| T651 | Sacrifice of tendon |
| T652 | Excision of lesion of tendon |
| T658 | Other specified excision of tendon |
| T659 | Unspecified excision of tendon |
| T67 | Primary repair of tendon |
| T671 | Primary repair of tendon using tendon transfer procedure |
| T672 | Primary repair of tendon using lengthening procedure |
| T673 | Primary repair of tendon using permanent prosthesis |
| T674 | Primary repair of tendon using temporary prosthesis |
| T675 | Primary repair of tendon using graft |
| T676 | Primary simple repair of tendon |
| T678 | Other specified primary repair of tendon |
| T679 | Unspecified primary repair of tendon |
| T68 | Secondary repair of tendon |
| T681 | Secondary repair of tendon using tendon transfer procedure |
| T682 | Secondary repair of tendon using lengthening procedure |
| T683 | Secondary repair of tendon using permanent prosthesis |
| T684 | Secondary repair of tendon using temporary prosthesis |
| T685 | Secondary repair of tendon using graft |
| T686 | Secondary simple repair of tendon |
| T688 | Other specified secondary repair of tendon |
| T689 | Unspecified secondary repair of tendon |
| T701 | Subcutaneous tenotomy |
| T702 | Tenotomy NEC |
| T74 | Other operations on tendon |
| T741 | Biopsy of lesion of tendon NEC |
| T742 | Removal of prosthesis from tendon |
| T743 | Exploration of tendon NEC |
| T744 | Injection of therapeutic substance into tendon NEC |
| T745 | Extracorporeal shockwave lithotripsy of calculus of tendon |
| T746 | Autologous blood injection into tendon |
| T747 | Injection of stem cells into tendon |
| T748 | Other specified other operations on tendon |
| T749 | Unspecified other operations on tendon |
| T774 | Debridement of muscle NEC |
| T962 | Excision of lesion of soft tissue NEC |
| T963 | Debridement of soft tissue NEC |
| T964 | Evacuation of seroma from soft tissue |
| T968 | Other specified other operations on soft tissue |
| T969 | Unspecified other operations on soft tissue |
| W054 | Attention to massive endoprosthesis of bone |
| W055 | Attention to endoprosthesis of bone NEC |
| W07 | Excision of ectopic bone |
| W071 | Excision of cross union of bone |
| W072 | Excision of periarticular ectopic bone |
| W073 | Excision of intramuscular ectopic bone |
| W078 | Other specified excision of ectopic bone |
| W079 | Unspecified excision of ectopic bone |
| W08 | Other excision of bone |
| W081 | Excision of natural protuberance of bone |
| W082 | Excision of overgrowth of bone |
| W083 | Excision of excrescence of bone |
| W084 | Excision of fragment of bone |
| W085 | Partial excision of bone NEC |
| W086 | Disarticulation of bone |
| W087 | Excision of accessory ossicle |
| W088 | Other specified other excision of bone |
| W089 | Unspecified other excision of bone |
| W09 | Extirpation of lesion of bone |
| W091 | Excision of lesion of bone NEC |
| W092 | Curettage of lesion of bone and graft HFQ |
| W093 | Curettage of lesion of bone NEC |
| W094 | Destruction of lesion of bone NEC |
| W095 | Curettage of tumour of bone and graft HFQ |
| W096 | Curettage of tumour of bone NEC |
| W097 | Excision of tumour of bone |
| W098 | Other specified extirpation of lesion of bone |
| W099 | Unspecified extirpation of lesion of bone |
| W178 | Other specified other reconstruction of bone |
| W179 | Unspecified other reconstruction of bone |
| W18 | Drainage of bone |
| W181 | Fenestration of cortex of bone |
| W182 | Saucerisation of bone |
| W183 | Sequestrectomy of bone |
| W184 | Decompression of fourage of bone |
| W185 | Insertion of drainage system into bone |
| W186 | Removal of drainage system from bone |
| W188 | Other specified drainage of bone |
| W189 | Unspecified drainage of bone |
| W191 | Primary open reduction of fracture of neck of femur and open fixation using pin and plate |
| W192 | Primary open reduction of fracture of long bone and fixation using rigid nail NEC |
| W193 | Primary open reduction of fracture of long bone and fixation using flexible nail |
| W194 | Primary open reduction of fracture of short bone and fixation using screw |
| W195 | Primary open reduction of fragment of bone and fixation using screw |
| W196 | Primary open reduction of fragment of bone and fixation using wire system |
| W198 | Other specified primary open reduction of fracture of bone and intramedullary fixation |
| W199 | Unspecified primary open reduction of fracture of bone and intramedullary fixation |
| W201 | Primary open reduction of fracture of long bone and extramedullary fixation using plate NEC |
| W202 | Primary open reduction of fracture of long bone and extramedullary fixation using cerclage |
| W203 | Primary open reduction of fracture of long bone and extramedullary fixation using suture |
| W204 | Primary open reduction of fracture of long bone and complex extramedullary fixation NEC |
| W208 | Other specified primary open reduction of fracture of bone and extramedullary fixation |
| W209 | Unspecified primary open reduction of fracture of bone and extramedullary fixation |
| W21 | Primary open reduction of intra-articular fracture of bone |
| W211 | Primary reduction of intra-articular fracture of bone using arthrotomy as approach |
| W212 | Primary excision of intra-articular fragment of intra-articular fracture of bone |
| W213 | Primary fixation of fragment of chondral cartilage of intra-articular fracture of bone |
| W214 | Primary intra-articular fixation of intra-articular fracture of bone NEC |
| W215 | Primary extra-articular reduction of intra-articular fracture of bone |
| W218 | Other specified primary open reduction of intra-articular fracture of bone |
| W219 | Unspecified primary open reduction of intra-articular fracture of bone |
| W22 | Other primary open reduction of fracture of bone |
| W221 | Primary open reduction of fracture of bone and skeletal traction HFQ |
| W222 | Primary open reduction of fracture of bone and external fixation HFQ |
| W228 | Other specified other primary open reduction of fracture of bone |
| W229 | Unspecified other primary open reduction of fracture of bone |
| W23 | Secondary open reduction of fracture of bone |
| W231 | Secondary open reduction of fracture of bone and intramedullary fixation HFQ |
| W232 | Secondary open reduction of fracture of bone and extramedullary fixation HFQ |
| W233 | Secondary open reduction of intra-articular fracture of bone |
| W234 | Secondary open reduction of fracture of bone and skeletal traction HFQ |
| W235 | Secondary open reduction of fracture of bone and external fixation HFQ |
| W236 | Secondary open reduction of fracture of bone and internal fixation HFQ |
| W238 | Other specified secondary open reduction of fracture of bone |
| W239 | Unspecified secondary open reduction of fracture of bone |
| W242 | Closed reduction of fracture of long bone and rigid internal fixation NEC |
| W243 | Closed reduction of fracture of long bone and flexible internal fixation HFQ |
| W244 | Closed reduction of fracture of short bone and fixation using screw |
| W245 | Closed reduction of fragment of bone and fixation using screw |
| W246 | Closed reduction of fracture of bone and fixation using nail or screw |
| W247 | Closed reduction of fracture of bone and fixation using plate |
| W248 | Other specified closed reduction of fracture of bone and internal fixation |
| W249 | Unspecified closed reduction of fracture of bone and internal fixation |
| W25 | Closed reduction of fracture of bone and external fixation |
| W251 | Closed reduction of fracture of bone and fixation to skeleton HFQ |
| W252 | Closed reduction of fracture of bone and fixation using functional bracing system |
| W253 | Remanipulation of fracture of bone and external fixation HFQ |
| W258 | Other specified closed reduction of fracture of bone and external fixation |
| W259 | Unspecified closed reduction of fracture of bone and external fixation |
| W26 | Other closed reduction of fracture of bone |
| W261 | Manipulation of fracture of bone and skeletal traction NEC |
| W262 | Manipulation of fracture of bone NEC |
| W263 | Remanipulation of fracture of bone and skeletal traction NEC |
| W264 | Remanipulation of fracture of bone NEC |
| W268 | Other specified other closed reduction of fracture of bone |
| W269 | Unspecified other closed reduction of fracture of bone |
| W28 | Other internal fixation of bone |
| W281 | Application of internal fixation to bone NEC |
| W282 | Adjustment to internal fixation of bone NEC |
| W283 | Removal of internal fixation from bone NEC |
| W284 | Insertion of intramedullary fixation and cementing of bone |
| W288 | Other specified other internal fixation of bone |
| W289 | Unspecified other internal fixation of bone |
| W29 | Skeletal traction of bone |
| W291 | Application of skeletal traction to bone NEC |
| W292 | Adjustment to skeletal traction of bone |
| W293 | Removal of skeletal traction from bone |
| W298 | Other specified skeletal traction of bone |
| W299 | Unspecified skeletal traction of bone |
| W30 | Other external fixation of bone |
| W301 | Application of external fixation to bone NEC |
| W302 | Adjustment to external fixation of bone NEC |
| W303 | Removal of external fixation from bone NEC |
| W304 | Application of external ring fixation to bone NEC |
| W308 | Other specified other external fixation of bone |
| W309 | Unspecified other external fixation of bone |
| W31 | Other autograft of bone |
| W311 | Inlay autograft to cortex of bone |
| W312 | Onlay autograft to cortex of bone |
| W313 | Cancellous strip autograft of bone |
| W314 | Cancellous chip autograft of bone |
| W315 | Vascularised pedicle autograft of bone |
| W316 | Muscle pedicle autograft of bone |
| W317 | Bone tendon autograft of bone |
| W318 | Other specified other autograft of bone |
| W319 | Unspecified other autograft of bone |
| W32 | Other graft of bone |
| W321 | Prepared graft of bone |
| W322 | Allograft of bone NEC |
| W323 | Xenograft of bone |
| W324 | Synthetic graft of bone |
| W325 | Cancellous chip allograft of bone |
| W326 | Bulk allograft of bone |
| W328 | Other specified other graft of bone |
| W329 | Unspecified other graft of bone |
| W33 | Other open operations on bone |
| W331 | Open biopsy of lesion of bone |
| W332 | Debridement of open fracture of bone |
| W333 | Suture of periosteum |
| W334 | Implantation of electromagnetic stimulator into bone |
| W335 | Attention to electromagnetic stimulator in bone |
| W336 | Debridement of bone NEC |
| W337 | Lavage of bone |
| W338 | Other specified other open operations on bone |
| W339 | Unspecified other open operations on bone |
| W35 | Therapeutic puncture of bone |
| W351 | Introduction of therapeutic substance into bone |
| W352 | Introduction of destructive substance into bone |
| W353 | Removal of implanted substance from bone |
| W354 | Therapeutic drilling of bone NEC |
| W355 | Therapeutic percutaneous puncture of bone |
| W356 | Percutaneous radiofrequency ablation of lesion of bone |
| W358 | Other specified therapeutic puncture of bone |
| W359 | Unspecified therapeutic puncture of bone |
| W361 | Percutaneous needle biopsy of lesion of bone |
| W362 | Needle biopsy of lesion of bone NEC |
| W363 | Diagnostic drilling of bone |
| W365 | Diagnostic extraction of bone marrow NEC |
| W368 | Other specified diagnostic puncture of bone |
| W369 | Unspecified diagnostic puncture of bone |
| W396 | Closed reduction of dislocated total prosthetic replacement of hip joint |
| W454 | Attention to total prosthetic replacement of joint NEC |
| W485 | Closed reduction of dislocated prosthetic replacement of head of femur |
| W544 | Attention to prosthetic replacement of articulation of bone NEC |
| W65 | Primary open reduction of traumatic dislocation of joint |
| W651 | Primary open reduction of fracture dislocation of joint and skeletal traction HFQ |
| W652 | Primary open reduction of traumatic dislocation of joint and skeletal traction NEC |
| W653 | Primary open reduction of fracture dislocation of joint NEC |
| W654 | Primary open reduction of fracture dislocation of joint and internal fixation NEC |
| W655 | Primary open reduction of fracture dislocation of joint and combined internal and external fixation |
| W658 | Other specified primary open reduction of traumatic dislocation of joint |
| W659 | Unspecified primary open reduction of traumatic dislocation of joint |
| W66 | Primary closed reduction of traumatic dislocation of joint |
| W661 | Primary closed reduction of fracture dislocation of joint and skeletal traction HFQ |
| W662 | Primary closed reduction of traumatic dislocation of joint and skeletal traction NEC |
| W663 | Primary manipulative closed reduction of fracture dislocation of joint NEC |
| W664 | Primary closed reduction of fracture dislocation of joint and internal fixation |
| W668 | Other specified primary closed reduction of traumatic dislocation of joint |
| W669 | Unspecified primary closed reduction of traumatic dislocation of joint |
| W67 | Secondary reduction of traumatic dislocation of joint |
| W671 | Secondary open reduction of fracture dislocation of joint and skeletal traction HFQ |
| W672 | Secondary open reduction of traumatic dislocation of joint and skeletal traction NEC |
| W673 | Secondary open reduction of fracture dislocation of joint NEC |
| W674 | Secondary open reduction of traumatic dislocation of joint NEC |
| W675 | Remanipulation of fracture dislocation of joint |
| W676 | Remanipulation of traumatic dislocation of joint |
| W677 | Secondary open reduction of fracture dislocation of joint and internal fixation NEC |
| W678 | Other specified secondary reduction of traumatic dislocation of joint |
| W679 | Unspecified secondary reduction of traumatic dislocation of joint |
| W69 | Open operations on synovial membrane of joint |
| W691 | Total synovectomy |
| W692 | Subtotal synovectomy |
| W693 | Partial synovectomy |
| W694 | Open biopsy of synovial membrane of joint |
| W695 | Open division of synovial plica |
| W698 | Other specified open operations on synovial membrane of joint |
| W699 | Unspecified open operations on synovial membrane of joint |
| W712 | Open excision of intra-articular osteophyte |
| W718 | Other specified other open operations on intra-articular structure |
| W719 | Unspecified other open operations on intra-articular structure |
| W72 | Prosthetic replacement of ligament |
| W721 | Primary prosthetic replacement of multiple ligaments |
| W722 | Prosthetic replacement of multiple ligaments NEC |
| W723 | Primary prosthetic replacement of intra-articular ligament |
| W724 | Prosthetic replacement of intra-articular ligament NEC |
| W725 | Primary prosthetic replacement of extra-articular ligament |
| W726 | Prosthetic replacement of extra-articular ligament NEC |
| W728 | Other specified prosthetic replacement of ligament |
| W729 | Unspecified prosthetic replacement of ligament |
| W73 | Prosthetic reinforcement of ligament |
| W731 | Primary extra-articular prosthetic augmentation of intra-articular ligament NEC |
| W732 | Extra-articular prosthetic augmentation of intra-articular ligament NEC |
| W733 | Primary prosthetic reinforcement of intra-articular ligament NEC |
| W734 | Prosthetic reinforcement of intra-articular ligament NEC |
| W738 | Other specified prosthetic reinforcement of ligament |
| W739 | Unspecified prosthetic reinforcement of ligament |
| W74 | Other reconstruction of ligament |
| W741 | Reconstruction of multiple ligaments NEC |
| W742 | Reconstruction of intra-articular ligament NEC |
| W743 | Reconstruction of extra-articular ligament NEC |
| W748 | Other specified other reconstruction of ligament |
| W749 | Unspecified other reconstruction of ligament |
| W75 | Other open repair of ligament |
| W751 | Open repair of multiple ligaments NEC |
| W752 | Open repair of intra-articular ligament NEC |
| W753 | Open repair of extra-articular ligament NEC |
| W758 | Other specified other open repair of ligament |
| W759 | Unspecified other open repair of ligament |
| W76 | Other operations on ligament |
| W761 | Excision of ligament |
| W762 | Excision of lesion of ligament |
| W763 | Biopsy of lesion of ligament |
| W768 | Other specified other operations on ligament |
| W769 | Unspecified other operations on ligament |
| W77 | Stabilising operations on joint |
| W771 | Repair of capsule of joint for stabilisation of joint NEC |
| W772 | Transposition of muscle for stabilisation of joint |
| W773 | Blocking operations on joint using prosthesis for stabilisation of joint |
| W774 | Blocking operations on joint using bone for stabilisation of joint |
| W775 | Periarticular osteotomy for stabilisation of joint |
| W776 | Annular ligament reconstruction for stabilisation of joint |
| W777 | Transposition of ligament for stabilisation of joint |
| W778 | Other specified stabilising operations on joint |
| W779 | Unspecified stabilising operations on joint |
| W782 | Release of contracture of hip joint |
| W784 | Limited release of contracture of capsule of joint |
| W788 | Other specified release of contracture of joint |
| W789 | Unspecified release of contracture of joint |
| W80 | Debridement and irrigation of joint |
| W801 | Open debridement and irrigation of joint |
| W802 | Open debridement of joint NEC |
| W803 | Open irrigation of joint NEC |
| W808 | Other specified debridement and irrigation of joint |
| W809 | Unspecified debridement and irrigation of joint |
| W81 | Other open operations on joint |
| W811 | Excision of lesion of joint NEC |
| W812 | Open removal of loose body from joint |
| W813 | Drainage of joint |
| W814 | Incision of joint NEC |
| W815 | Exploration of joint NEC |
| W816 | Capsulorrhaphy of joint |
| W817 | Insertion of therapeutic spacer into joint |
| W818 | Other specified other open operations on joint |
| W819 | Unspecified other open operations on joint |
| W843 | Endoscopic division of synovial plica |
| W844 | Endoscopic decompression of joint |
| W846 | Endoscopic excision of synovial plica |
| W848 | Other specified therapeutic endoscopic operations on other joint structure |
| W849 | Unspecified therapeutic endoscopic operations on other joint structure |
| W86 | Therapeutic endoscopic operations on cavity of other joint |
| W861 | Endoscopic removal of loose body from joint NEC |
| W868 | Other specified therapeutic endoscopic operations on cavity of other joint |
| W869 | Unspecified therapeutic endoscopic operations on cavity of other joint |
| W88 | Diagnostic endoscopic examination of other joint |
| W881 | Diagnostic endoscopic examination of joint and biopsy of lesion of joint NEC |
| W888 | Other specified diagnostic endoscopic examination of other joint |
| W889 | Unspecified diagnostic endoscopic examination of other joint |
| W90 | Puncture of joint |
| W901 | Aspiration of joint |
| W902 | Arthrography |
| W903 | Injection of therapeutic substance into joint |
| W904 | Injection into joint NEC |
| W908 | Other specified puncture of joint |
| W909 | Unspecified puncture of joint |
| W911 | Manipulation of joint using traction NEC |
| W913 | Manipulation of prosthetic joint NEC |
| W918 | Other specified other manipulation of joint |
| W919 | Unspecified other manipulation of joint |
| W92 | Other operations on joint |
| W921 | Biopsy of lesion of joint NEC |
| W922 | Distension of joint |
| W923 | Examination of joint under image intensifier |
| W924 | Examination of joint under anaesthetic |
| W925 | Examination of joint NEC |
| W926 | Chemical synovectomy |
| W927 | Radiation synovectomy |
| W928 | Other specified other operations on joint |
| W929 | Unspecified other operations on joint |
| X091 | Hindquarter amputation |
| X092 | Disarticulation of hip |
| X098 | Other specified amputation of leg |
| X099 | Unspecified amputation of leg |
| X12 | Operations on amputation stump |
| X121 | Reamputation at higher level |
| X122 | Excision of lesion of amputation stump |
| X123 | Shortening of length of amputation stump |
| X124 | Revision of coverage of amputation stump |
| X125 | Drainage of amputation stump |
| X128 | Other specified operations on amputation stump |
| X129 | Unspecified operations on amputation stump |
