# Supplementary material for: Social inequalities in patient outcomes after total hip replacement surgery for osteoarthritis in England: A population-based cohort study of the National Joint Registry
Source: PLoS Med. 2026 Feb 2;23(2):e1004870. doi: 10.1371/journal.pmed.1004870 (PMC12863669; doi:10.1371/journal.pmed.1004870)
Supplement: S5 Table — (DOCX) [file pmed.1004870.s009.docx]

S5 Table: Outcomes for patients following primary hip replacement in England for patients with linked HES or PROMs data by Index of Multiple Deprivation (IMD) (2007 to 2017)

|  |  |  |  |  | IMD | | | | | | | | | |  |  |  |  |
| --- | --- | --- | --- | --- | --- | --- | --- | --- | --- | --- | --- | --- | --- | --- | --- | --- | --- | --- |
|  |  | **All** |  |  | **Q5**  **Least deprived** | | **Q4** | | **Q3** | | **Q2** | | **Q1**  **Most deprived** | | **Unadjusted rate ratio#** | **Unadjusted risk difference#** | **Unadjusted NNTH#** | **Adjusted NNTH#*** |
|  | **N** | **Cases** | **Rate**  **%** |  | **Cases** | **Rate**  **%** | **Cases** | **Rate**  **%** | **Cases** | **Rate**  **%** | **Cases** | **Rate**  **%** | **Cases** | **Rate**  **%** | **[95%CI]** | **% [95%CI]** | **[95%CI]** | **[95%CI]** |
| Cumulative mortality by 90 days | 448,184 | 1,695 | 0.38 |  | 379 | 0.36 | 394 | 0.36 | 353 | 0.35 | 307 | 0.40 | 262 | 0.47 | 1.31 [1.12, 1.54] | 0.11 [0.05, 0.18] | 896 [560, 2,239] | 1,105 [640, 4,044] |
| Cumulative revisions by 5 years | 448,184 | 7,023 | 1.57 |  | 1,612 | 1.52 | 1,716 | 1.58 | 1,561 | 1.55 | 1,216 | 1.59 | 918 | 1.64 | 1.08 [1.00, 1.17] | 0.12 [0, 0.25] | 807 [397, ∞]^ⴕ^ | 4,123 [662, ∞]^ⴕ^ |
| Cumulative complications by 6 months | 448,184 | 21,527 | 4.8 |  | 4,567 | 4.29 | 5,009 | 4.61 | 4,803 | 4.77 | 3,938 | 5.15 | 3,210 | 5.73 | 1.34 [1.28, 1.40] | 1.44 [1.21, 1.67] | 69 [60, 83] | 88 [74, 109] |
| Cumulative rehospitalisations by 1 year | 448,184 | 78,968 | 17.62 |  | 17,714 | 16.65 | 18,442 | 16.98 | 17,499 | 17.38 | 13,999 | 18.29 | 11,314 | 20.20 | 1.21 [1.19, 1.24] | 3.55 [3.15, 3.95] | 28 [25, 32] | 36 [31, 42] |
| Cumulative reoperations by 1 year | 448,184 | 6,637 | 1.48 |  | 1,441 | 1.35 | 1,528 | 1.41 | 1,451 | 1.44 | 1,229 | 1.61 | 988 | 1.76 | 1.30 [1.20, 1.41] | 0.41 [0.28, 0.54] | 244 [186, 357] | 319 [227, 539] |
| Oxford Hip Score MCID~ | 200,522 | 10,647 | 5.31 |  | 2,359 | 4.65 | 2,345 | 4.67 | 2,307 | 5.10 | 1,916 | 5.88 | 1,720 | 7.91 | 1.70 [1.60, 1.81] | 3.26 [2.86, 3.67] | 31 [27, 35] | 33 [29, 38] |

# Most deprived (Q1) versus least deprived (Q5: Reference)

*Adjusted for adjusted for sex, age group, body mass index, American Society of Anesthesiologists grade and Charlson score at primary operation

ⴕConfidence intervals are wide and include infinity to number needed to benefit: presented are the number needed to harm (NNTH) value (95% confidence interval, NNTH to ∞ to number needed to benefit [NNTB])

~MCID, Minimal Clinically Important Difference for the full Oxford Hip Score set at a five-point increase from pre- to post-total hip replacement operation (coded cases=’Not improved <5 points’)

Abbreviations: CI, Confidence Interval; IMD, Index of Multiple Deprivation; N, number; NNTB, Number needed to benefit; NNTH, Number needed to harm; MCID, Minimal Clinically Important Difference; PROMs Patient Reported Outcome Measures; Q, quintile
