# Supplementary material for: Social inequalities in patient outcomes after total hip replacement surgery for osteoarthritis in England: A population-based cohort study of the National Joint Registry
Source: PLoS Med. 2026 Feb 2;23(2):e1004870. doi: 10.1371/journal.pmed.1004870 (PMC12863669; doi:10.1371/journal.pmed.1004870)
Supplement: S6 Table — (DOCX) [file pmed.1004870.s010.docx]

S6 Table: Cumulative complications at 6 months post hip replacement by Index of Multiple Deprivation (IMD) group (N=448,184)

|  |  |  |  | IMD  N with complication | | | | | IMD  Rate per 10,000 patients | | | | | | Unadjusted rate ratio# | | Unadjusted risk difference# | | Unadjusted NNTH# | | Adjusted NNTH#* | |  |
| --- | --- | --- | --- | --- | --- | --- | --- | --- | --- | --- | --- | --- | --- | --- | --- | --- | --- | --- | --- | --- | --- | --- | --- |
|  | **N with complication** | **Overall rate per 10,000 patients** |  | **Q5 Least deprived** | **Q4** | **Q3** | **Q2** | **Q1 Most deprived** | | **Q5 Least deprived** | **Q4** | **Q3** | **Q2** | **Q1 Most deprived** | | **(95%CI]** | | **per 10,000**  **(95%CI]** | | **(95%CI]** | | **(95%CI]** | |
| Health-related complications |  |  |  |  |  |  |  |  | |  |  |  |  |  | |  | |  | |  | |  | |
| Urinary tract infection | 4,486 | 100 |  | 924 | 1,029 | 1,006 | 878 | 649 | | 87 | 95 | 100 | 115 | 116 | | 1.33 [1.21, 1.47] | | 29 [19, 40] | | 344 [253, 539] | | 407 [286, 700] | |
| Respiratory infection | 4,411 | 98 |  | 826 | 988 | 940 | 851 | 806 | | 78 | 91 | 93 | 111 | 144 | | 1.85 [1.68, 2.04] | | 66 [55, 78] | | 151 [129, 182] | | 185 [155, 231] | |
| Acute renal failure | 3,207 | 72 |  | 664 | 735 | 695 | 622 | 491 | | 62 | 68 | 69 | 81 | 88 | | 1.40 [1.25, 1.58] | | 25 [16, 34] | | 396 [291, 617] | | 539 [365, 1,027] | |
| Acute myocardial infarction | 1,506 | 34 |  | 358 | 326 | 333 | 268 | 221 | | 34 | 30 | 33 | 35 | 39 | | 1.17 [0.99, 1.39] | | 6 [0, 12] | | 1,721 [829, ∞]^ⴕ^ | | 1,870 [859, ∞]^ⴕ^ | |
| Stroke | 1,236 | 28 |  | 227 | 308 | 278 | 235 | 188 | | 21 | 28 | 28 | 31 | 34 | | 1.57 [1.30, 1.91] | | 12 [7, 18] | | 818 [563, 1,493] | | 856 [582, 1,620] | |
| Any health-related complications | 12,093 | 270 |  | 2,469 | 2,744 | 2,660 | 2,303 | 1,917 | | 232 | 253 | 264 | 301 | 342 | | 1.47 [1.39, 1.56] | | 110 [93, 128] | | 91 [78, 108] | | 110 [92, 135] | |
| Surgery-related complications |  |  |  |  |  |  |  |  | |  |  |  |  |  | |  | |  | |  | |  | |
| Prothesis complication | 4,992 | 111 |  | 1,132 | 1,184 | 1,107 | 863 | 706 | | 106 | 109 | 110 | 113 | 126 | | 1.18 [1.08, 1.30] | | 20 [9, 31] | | 509 [325, 1,170] | | 615 [366, 1,936] | |
| Pulmonary embolism/deep vein thrombosis | 2,399 | 54 |  | 524 | 606 | 522 | 423 | 324 | | 49 | 56 | 52 | 55 | 58 | | 1.17 [1.02, 1.35] | | 9 [1, 16] | | 1,163 [619, 9,639] | | 1,344 [665, ∞]^ⴕ^ | |
| Surgical site infection | 2,316 | 52 |  | 457 | 530 | 558 | 416 | 355 | | 43 | 49 | 55 | 54 | 63 | | 1.48 [1.29, 1.69] | | 20 [13, 28] | | 489 [356, 783] | | 699 [460, 1,456] | |
| Wound disruption | 902 | 20 |  | 186 | 213 | 186 | 167 | 150 | | 17 | 20 | 18 | 22 | 27 | | 1.53 [1.24, 1.90] | | 9 [4, 14] | | 1,075 [701, 2,305] | | 1,347 [816, 3,859] | |
| Blood transfusion | 638 | 14 |  | 152 | 168 | 146 | 107 | 65 | | 14 | 15 | 14 | 14 | 12 | | 0.81 [0.61, 1.09] | | -3 [-6, 1] | | NNTB 3,729 [∞, NNTB 1,587]^ⴕ^ | | NNTB 2,674 [NNTB 1,369, NNTB 58,324] | |
| Fracture after implant | 580 | 13 |  | 117 | 146 | 142 | 95 | 80 | | 11 | 13 | 14 | 12 | 14 | | 1.30 [0.98, 1.73] | | 3 [0, 7] | | 3,042 [1,429, ∞]^ⴕ^ | | 3,565 [1,536, ∞]^ⴕ^ | |
| Neurovascular injury | 92 | 2 |  | 19 | 22 | 26 | 15 | 10 | | 2 | 2 | 3 | 2 | 2 | | 1.00 [0.46, 2.15] | | 0 [-1, 1] | | 0 [∞, NNTB 7,311]^ⴕ^ | | NNTB 245,956 [∞, NNTB 7,164]^ⴕ^ | |
| Any surgery-related complications | 11,135 | 248 |  | 2,426 | 2,681 | 2,513 | 1,943 | 1,572 | | 228 | 247 | 250 | 254 | 281 | | 1.23 [1.16, 1.31] | | 53 [36, 69] | | 190 [145, 276] | | 253 [179, 427] | |

# Most deprived (Q1) versus least deprived (Q5: Reference)

*Adjusted for adjusted for sex, age group, body mass index, American Society of Anesthesiologists grade and Charlson score at primary operation

ⴕConfidence intervals are wide and include infinity to number needed to benefit: presented are the number needed to harm (NNTH) value (95% confidence interval, NNTH to ∞ to number needed to benefit [NNTB])

Abbreviations: CI, Confidence Interval; IMD, Index of Multiple Deprivation; N, number; NNTB, Number needed to benefit; NNTH, Number needed to harm; MCID, Minimal Clinically Important Difference; Q, quintile
